# Revolutionizing Tetracycline Hydrochloride Remediation: 3D Motile Light‐Driven MOFs Based Micromotors in Harsh Saline Environments

**DOI:** 10.1002/advs.202406381

**Published:** 2024-08-29

**Authors:** Yu Zhao, Jiawei Lin, Qing Wu, Yulong Ying, Josep Puigmartí‐Luis, Salvador Pané, Sheng Wang

**Affiliations:** ^1^ School of Materials Science and Engineering Zhejiang Sci‐Tech University Hangzhou 310018 P. R. China; ^2^ Departament de Ciència dels Materials i Química Física Institut de Química Teòrica i Computacional University of Barcelona Martí i Franquès, 1 Barcelona 08028 Spain; ^3^ Institució Catalana de Recerca i Estudis Avançats (ICREA) Pg. Lluís Companys 23 Barcelona 08010 Spain; ^4^ Multi‐Scale Robotics Lab Institute of Robotics and Intelligent Systems ETH Zurich Tannenstrasse 3 Zurich 8092 Switzerland

**Keywords:** harsh saline environment, light‐driven, metal‐organic frameworks, micromotors, tetracycline hydrochloride remediation

## Abstract

Traditional light‐driven metal‐organic‐frameworks (MOFs)‐based micromotors (MOFtors) are typically constrained to two‐dimensional (2D) motion under ultraviolet or near‐infrared light and often demonstrate instability and susceptibility to ions in high‐saline environments. This limitation is particularly relevant to employing micromotors in water purification, as real wastewater is frequently coupled with high salinity. In response to these challenges, ultrastable MOFtors capable of three‐dimensional (3D) motion under a broad spectrum of light through thermophoresis and electrophoresis are successfully synthesized. The MOFtors integrated photocatalytic porphyrin MOFs (PCN‐224) with a photothermal component made of polypyrrole (PPy) by three distinct methodologies, resulting in micromotors with different motion behavior and catalytic performance. Impressively, the optimized MOFtors display exceptional maximum velocity of 1305 ± 327 µm s^−1^ under blue light and 2357 ± 453 µm s^−1^ under UV light. In harsh saline environments, these MOFtors are not only maintain high motility but also exhibit superior tetracycline hydrochloride (TCH) removal efficiency of 3578 ± 510 mg g^−1^, coupling with sulfate radical‐based advanced oxidation processes and peroxymonosulfate. This research underscores the significant potential of highly efficient MOFtors with robust photocatalytic activity in effectively removing TCH in challenging saline conditions, representing a substantial advancement in applying MOFtors within real‐world water treatment technologies.

## Graphical abstract

This study developed light‐driven three‐dimension motile polypyrrole (PPy)‐modified porphyrin (PCN)‐based micromotors (MOFtors) based on thermophoresis and electrophoresis. By incorporating PPy through three different methods, its motion and photocatalytic performance are optimized. In high‐saline environments, these MOFtors maintained high mobility and effectively removed tetracycline hydrochloride, offering a cost‐effective solution for practical water treatment and representing the substantial advancement of micro/nanomotors.

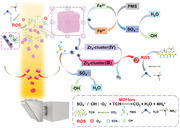

## 1 Introduction

Over the past century, antibiotics have played a pivotal role in human healthcare, animal husbandry, and aquaculture, significantly contributing to controlling infectious diseases.^[^ ^1^ ^]^ Among these, tetracycline hydrochloride (TCH) stands out as a widely utilized broad‐spectrum antibiotic, known for its efficacy and cost‐effectiveness. However, the extensive use of TCH has led to the accumulation of its residues and metabolites in aquatic environments, a scenario that has emerged in recent years.^[^ ^2^ ^]^ The persistence of TCH residues in water poses considerable risks to both human health and ecological systems, owing to their ecotoxicity and resistance to biodegradation.^[^ ^3^ ^]^ This alarming situation underscores the urgent need to develop efficient and eco‐friendly technologies for TCH degradation, addressing the critical environmental challenge of our times.

At present, conventional wastewater treatment methods, including adsorption,^[^ ^4^ ^]^ electrochemical oxidation,^[^ ^5^ ^]^ coagulation,^[^ ^6^ ^]^ and membrane separation,^[^ ^7^ ^]^ face significant challenges in effectively removing TCH residues from the environment. Advanced oxidation processes (AOPs) are considered the most promising TCH removal approach because of their simplicity, high efficiency, and reproducibility.^[^ ^8^ ^]^ AOPs facilitate the in situ generation of highly active free radicals (i.e., hydroxyl radical (•OH), superoxide anion radical (•O_2_^−^), sulfate (SO_4_^•−^)) through chemical, photochemical, and electrochemical processes, thereby decomposing organic pollutants.^[^ ^9^ ^]^ Sulfate radical‐based advanced oxidation processes (SR‐AOPs) offer a highly effective strategy for treating organic pollutants. SR‐AOPs offer several advantages over traditional •OH‐based AOPs, including a higher oxidation potential (SO_4_^•−^, 2.6–3.1 V vs •OH, 1.8–2.7 V), a broader operational pH range (2–8), and a longer half‐life (SO_4_^•−^, 30–40 µs vs •OH, <1 µs).^[^ ^10^ ^]^ The short lifespan of free radicals presents a challenge, as they are quickly quenched if they are not in immediate interaction with contaminants. Additionally, the limited penetration depth of light, particularly within ultra‐violet spectrum, which is essential for photocatalysis, requires mechanical stirring to transport the catalyst to the surface and ensure its exposure to light.

Recently, micro/nanomotors (MNMs) have emerged as potential tools in wastewater treatment, proving particularly effective in environments where mechanical stirring is unavailable or unfeasible.^[^ ^11^ ^]^ MNMs display self‐propulsion abilities through various power sources, such as chemical fuels (hydrogen peroxide, urea, etc.),^[^ ^12^ ^]^ electric,^[^ ^13^ ^]^ magnetic,^[^ ^14^ ^]^ acoustics fields, or light.^[^ ^15^ ^]^ Self‐propulsion facilitates fluid mix around contaminated samples, thereby minimizing transport by diffusion and ultimately accelerating pollutant removal.^[^ ^16^ ^]^ Among these power sources, light is increasingly favored for driving MNMs because of its remote controllability, non‐contact nature, non‐pollution characteristics, and cost‐effectiveness.^[^ ^17^ ^]^ However, deploying light‐driven MNMs in harsh saline environments, which are common for TCH‐contaminated water, poses significant challenges. These environments present unique obstacles to the effective operation of MNMs, as salinity can impact both the mechanical and chemical dynamics essential for their optimal functionality.^[^ ^18^ ^]^ Addressing this issue is crucial for expanding the applicability of light‐driven MNMs in real‐world wastewater treatment scenarios.

Metal‐organic frameworks (MOFs) have gained widespread attention in various fields, including absorption and separation,^[^ ^19^ ^]^ catalysis,^[^ ^20^ ^]^ drug delivery,^[^ ^21^ ^]^ and sensing,^[^ ^22^ ^]^ primarily due to their ultra‐high surface area, customizable porosity biocompatibility, and multiple active sites. As emerging catalysts, MOF‐based materials have been extensively utilized in SR‐AOPs, achieving many significant outcomes.^[^ ^23^ ^]^ MOF‐based micromotors (MOFtors) combine MOFs with MNMs, opening new avenues for applications in water pollution remediation. Light is an ideal external source for driving MOFtors because of its remote control over their motility, as well as its speed and on/off motion. Several MOFs display semiconductor‐like properties when exposed to light. Typically, organic ligands absorb light energy, which facilitate charge transfer from the ligands to the metal clusters. This process generates electron‐hole pairs, leading to photoredox reactions in the presence of the electrolyte and producing highly oxidizing free radicals.^[^ ^24^ ^]^ To engineer light‐driven MNMs, photocatalytic and photothermal materials are often integrated.^[^ ^17^ ^]^ Porphyrin‐based MOFs, in particular, are exceptional photothermal materials. They can induce photoelectrochemical reactions and photothermal conversion by harnessing light energy, thereby generating an asymmetric field for propulsion.^[^ ^25^ ^]^ This feature makes them highly suitable for realizing MOFtors. Particularly, PCN‐224, a Zr‐based porphyrin MOF, has excellent chemical stability and robust motion capability. It remains stable in a wide range of pH (pH = 0–12), both in aqueous and acidic‐alkaline solutions, and it retains its motion even in high salinity environments.^[^ ^25^, ^26^ ^]^ These capabilities are significant advantages over conventional light‐driven MNMs, which often struggle to maintain structure and motion in harsh environments.^[^ ^18b,c^ ^]^ The robustness of PCN‐224 enhances the feasibility of using light‐driven MOFtors in practical wastewater treatment scenarios, expanding their applicability in various challenging environmental conditions. Simultaneously, certain polymer materials exhibit both photocatalytic and photothermal properties, making them excellent candidates for fabricating light‐driven MNMs. Among them, polypyrrole (PPy) stands out as a suitable candidate for its high stability, high charge mobility, and abundance of photocatalytic reaction sites. Additionally, PPy has a broad light absorption range covering the entire ultraviolet‐visible spectrum.

Herein, we introduced MOFtors composed of a porphyrin‐based MOF (PCN‐224), characterized by its unique ring‐conjugated structure, coupled with a photoactive component made of PPy, for efficient TCH remediation.^[^ ^27^ ^]^ This integration allowed for both a robust three‐dimension (3D) motion capability and an active photodegradation performance when combined with potassium peroxymonosulfate (PMS)‐mediated SR‐AOPs in a challenging high‐saline environment. By incorporating photothermal PPy through three distinct routes, we can create three different variants of MOFtors with different movement behavior and catalytic performance. Under ultraviolet, visible, and near‐infrared light, both thermophoresis and electrophoresis were generated, enabling the 3D motion of MOFtors and facilitating effective TCH degradation. The autonomous motion of MOFtors enhances fluid mixing, thereby extending the range and effectiveness of short‐lived radicals. Additionally, the introduction of PMS into the system synergizes with SR‐AOPs, significantly improving the TCH degradation efficiency. Our results demonstrate that the maximum velocity of MOFtors can reach 1305 ± 327 µm s^−1^, and their optimal degradation capability achieves 3347 ± 302 mg g^−1^, significantly surpassing current state‐of‐the‐art approaches. We further evaluated TCH degradation in various saline solution environments, simulating real‐world water pollution scenarios. Our findings indicate that the degradation efficiency was largely ion‐independent, with the exception of Cl^−^ ions, where chlorine free radicals could be activated to further enhance the TCH degradation (3578 ± 510 mg g^−1^). The strategy of employing light‐driven, high‐ion‐tolerance MOFtors as active catalysts represents a novel paradigm for addressing the challenge of non‐biodegradable organic pollutants, showcasing not only the potential of MOFtors in practical water remediation applications but also the utilization of MNMs for environmental cleanup.

## 2 Results and Discussion

### 2.1 Synthesis and Characterization of PCN‐224 and PCN‐PPy Variants

**Figure 1:**
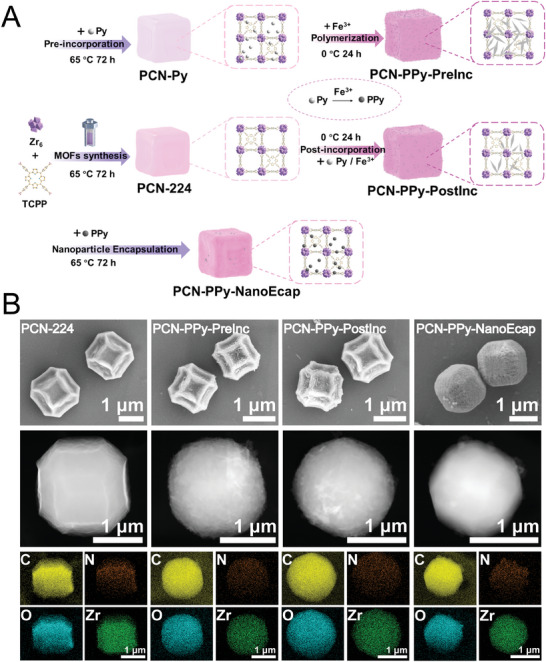
Preparation and morphological characterization of MOFtors. A) Schematic illustration of the preparation of PCN‐224 and PCN‐PPy variants based MOFtors. B) SEM and TEM images of PCN‐224 and PCN‐PPy variants and the corresponding elemental distribution mapping images.

PCN‐224 was synthesized by a simple solvothermal method following a previously reported method with minor modification.^[^ ^25^, ^28^ ^]^ Zirconium dichloride octahydrate (ZrOCl_2_.8H_2_O), 4,4,4,4‐(Porphine‐5,10,15,20‐tetrayl)tetrakis(benzoic acid) (H_2_TCPP) and acetic acid were dispersed in N,N dimethylformamide (DMF) solution and solvothermal reaction was carried out to obtain dark purple PCN‐224 crystals. The incorporation of PCN‐224 and polypyrrole (PPy) was prepared by three different methods, yielding three different products labeled as PCN‐224‐PreInc, PCN‐PPy‐PostInc, and PCN‐PPy‐NanoEncap (**Figure** **1**A).^[^ ^29^ ^]^ First, PCN‐PPy‐PreInc (Pre‐Incorporation) was synthesized by an in situ encapsulation, involving the incorporation of pyrrole monomers during the solvothermal synthesis of PCN‐224. Subsequently, the pyrrole was polymerized by ferric chloride (FeCl_3_.6H_2_O) as the polymerizing agent. PCN‐PPy‐PostInc (Post‐Incorporation) was prepared by impregnating first PCN‐224 with the pyrrole monomer and then polymerized following the same approach conducted for PCN‐PPy‐PreInc. PCN‐PPy‐NanoEncap (Nanoparticles‐Encapsulation) was prepared by a one‐pot solvothermal reaction, where PPy nanoparticles (NPs) were physically encapsulated within the growing structure of PCN‐224. This encapsulation occurred simultaneously with the growth process of PCN‐224, ensuring a cohesive integration of PPy NPs within the final product. As shown in Figure 1, the morphological characteristics of PCN‐224 and its variants (PCN‐PPy‐PreInc, PCN‐PPy‐PostInc, and PCN‐PPy‐NanoEncap) were analyzed using transmission electron microscope (TEM) and scanning electron microscope (SEM) imaging techniques. The successfully synthesized PCN‐224 exhibited a cubic shape with slightly concave edges and a relatively uniform size of ≈1.5 µm. The morphologies of both PCN‐PPy‐PreInc and PCN‐PPy‐PostInc closely resembled that of PCN‐224, with the noticeable presence of fibrous PPy structures visible in the aperture and on the surface of the MOFs. In contrast, PCN‐PPy‐NanoEncap displayed a distinctly full cubic shape, with spherical PPy NPs visibly embedded on its surface. The high‐magnification SEM images in Figure S1 (Supporting Information) revealed that the surface of the cubic structures was densely populated with PPy areas, either as fibrous extensions or spherical deposits, significantly altering the surface texture of the cubes. Further analysis was conducted on these three PCN‐PPy variants that underwent a grinding treatment. The cross‐sectional SEM images in Figure S2 (Supporting Information) revealed insightful details regarding the distribution of PPy within PCN‐224 structure and the interaction of PPy formations with PCN‐224 framework. High‐angle annular dark field transmission electron microscopy (HAADF‐STEM) images, along with corresponding elemental mapping analyses of PCN‐224 and PCN‐PPy variants, were shown in Figures 1C and S3 (Supporting Information), confirming the morphological differences among the specimens. The elemental mapping displayed the spatial distribution of carbon (C), nitrogen (N), oxygen (O), and zirconium (Zr). In the case of PCN‐PPy‐PreInc and PCN‐PPy‐PostInc, the presence of Fe element was attributed to the incorporation of FeCl_3_ residuals during the PPy polymerization process (as evidenced in Figure S3, Supporting Information). The presence of Fe was further confirmed by energy‐dispersive X‐ray spectrometer (EDS) spectrum (Figure S4, Supporting Information). In addition, local magnified transmission images of PCN‐PPy‐PreInc, PCN‐PPy‐PostInc, and PCN‐PPy‐NanoEncap in Figure S5 (Supporting Information) offered a vivid depiction of the internal structure of PPy within the PCN‐224 frameworks. Collectively, these images enhanced our understanding of the compositional and structural integration of PPy in the PCN‐224 matrix.

**Figure 2:**
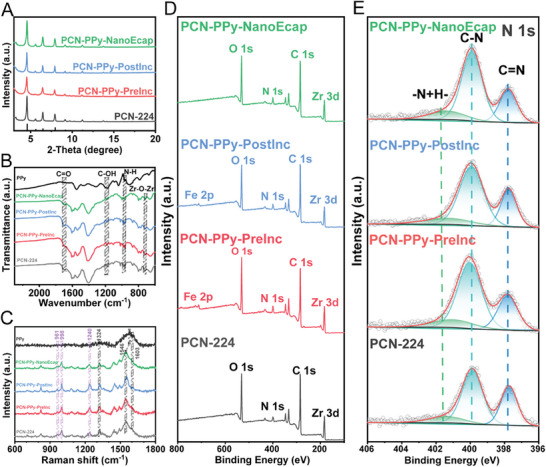
Structural characterizations. A) XRD patterns, B) FTIR spectra, C) Raman spectra, D) full XPS spectra of PCN‐224 and PCN‐PPy variants, and E) high‐resolution XPS spectra of N 1s for PCN‐224 and PCN‐PPy variants.

The X‐ray diffraction (XRD) patterns of PCN‐224 and its PCN‐PPy variants in **Figure** **2**A showed sharp characteristic peaks, consistent in both 2θ angles and intensities with simulated profiles. The peaks located at 4.6, 6.5, 7.9, and 9.1° correspond to (002), (022), (222), and (004) crystal planes of PCN‐224, respectively.^[^ ^30^ ^]^ Importantly, the incorporation of PPy through these three doping methods does not alter the inherent crystal structure of PCN‐224. Furthermore, because of the amorphous nature and relatively low PPy content, no additional diffraction peaks belonging to PPy were observed in the XRD patterns. Subsequently, samples were characterized by Fourier transform infrared spectroscopy (FTIR) (Figure 2B). The characteristic peaks at 967 and 1697 cm^−1^ are attributed to the N─H absorption vibration in H_2_TCPP and the C═O vibration of carboxylic acid groups, while the peak at 1182 cm^−1^ represents the asymmetric vibration peak of C─OH group.^[^ ^31^ ^]^ The band at 762 cm^−1^ corresponds to the wobble vibration of Zr─O─Zr in the zirconium porphyrin MOFs.^[^ ^32^ ^]^ The benzene ring vibration is confirmed by the peak ≈1546 cm^−1^.^[^ ^33^ ^]^ PCN‐224 and PCN‐PPy variants were further characterized by Raman spectroscopy. The most prominent peaks in the Raman spectra, observed at 1546 and 1573 cm^−1^ are indicative of the extension of C─C bond and the bending deformation of C─N(H)─C. The characteristic Raman signals at 1603 and 1324 cm^−1^ are associated with the bending deformation of the C─N(H)─C bond with the pyrrole ring. The strong Raman peaks of PCN‐224 and PCN‐PPy were observed at 1240 cm^−1^, which are related to the bending deformation within the phenyl group. This peak is absent in PPy due to the lack of a benzene ring structure. Additionally, the observed Raman peaks at 998 and 961 cm^−1^ correspond to in‐plane bending of the benzene ring.^[^ ^34^ ^]^ However, note that these spectral observations alone are not sufficient to conclusively prove the successful incorporation of PPy, because of the similarity in bonds within the pyrrole and the porphyrin structure.

To explore the chemical state, coordination environment, and elemental composition of PCN‐224 and its PCN‐PPy variants, X‐ray photoelectron spectroscopy (XPS) analyses were conducted to identify any differences between them. The XPS spectra in Figure 2D revealed the elemental compositions of those materials, confirming the presence of C, N, O, Zr, and Fe. These findings were consistent with the EDS results. A detailed examination was provided in Figure 2E, where the high‐resolution N 1s XPS spectrum shows three distinct three N species, including ─HN^+^─ (401.2 eV), ─CN─ (399.9 eV), and ─C═N─ (397.8 eV), respectively.^[^ ^35^ ^]^ In particular, the PCN‐PPy variants showed a higher total nitrogen content and a reduced C═N ratio compared with PCN‐224. For instance, the PCN‐PPy‐PreInc showed a nitrogen content of 5.71 at.%, and a decreased C═N ratio of 26.9%, indicating the successful incorporation of PPy (Tables S1 and S2, Supporting Information). The elemental compositions, particularly the Fe content in PCN‐PPy‐PreInc and PCN‐PPy‐PostInc, as determined from XPS spectra and presented in Table S2 (Supporting Information), were found to be 1.13 at.% and 0.9 at%, respectively. The incorporation of Fe ions within the PPy occurred inevitably during the polymerization process. Two prominent core doublets of Fe^2+^ were observed in the XPS spectra of Fe 2p for both PCN‐PPy‐PreInc and PCN‐PPy‐PostInc, appearing at 724.9 eV (Fe 2p^1/2^) and 711.2 eV (Fe 2p^3/2^). Similarly, doublets of Fe^3+^ at 728.5 eV (Fe 2p^1/2^) and 715.1 eV (Fe 2p^3/2^) were also detected for both samples. Both Fe^2+^ and Fe^3+^ doublets were companied with satellite peaks (Figure S6, Supporting Information). For Fe ions to coordinate with MOF, specific synthesis conditions are required, typically involving hydrothermal methods at elevated temperatures and the use of N,N‐dimethylformamide as a solvent.^[^ ^23^ ^]^ In our work, the synthesis was conducted at low temperature environment using pure water as the solvent, making it challenging for Fe ions to coordinate with the MOF. The surface area and pore distribution of all samples were analyzed using the N_2_ adsorption–desorption isotherm at 77 K, combined with the non‐local density functional theory (NLDFT) method (Figure S7, Supporting Information). PCN‐224 exhibited a type I isotherm, indicative of a microporous structure, with a BET surface area and total pore volume estimated to be 2592 m^2^ g^−1^ and 1.123 cm^3^ g^−1^, respectively. In comparison, the BET surface area and total pore volume of PCN‐PPy‐PreInc were, respectively, 2054 m^2^ g^−1^ and 0.908 cm^3^ g^−1^, while PCN‐PPy‐PostInc and PCN‐PPy‐NanoEncap displayed values of 1881 m^2^ g^−1^ and 0.324 cm^3^ g^−1^, and 515 m^2^ g^−1^ and 0.241 cm^3^ g^−1^, respectively. Notably, the isotherm for PCN‐PPy‐PostInc presented an H_4_‐type hysteresis loop, characteristic of a composite of type I and type II adsorption isotherms, suggesting the formation of additional mesopores within its structure. The pore size distribution analysis revealed a significant reduction in the average pore size within the micropore range following the incorporation of PPy. This reduction varied depending on the specific encapsulation method used for the PPy integration, which influenced the structure properties of the materials.

### 2.2 Motion Behavior Study

Porphyrins and their derivatives, regarded as excellent photosensitizers, have excellent photophysical properties, including strong absorption bands in the visible region. This makes porphyrin‐like organic units particularly attractive as components in MOFtors, as they can mimic natural photosynthetic systems. Concurrently, PPy is characterized by its wide‐band absorption, excellent conductivity, and photothermal effect across ultraviolet and near‐infrared/short‐wave infrared spectra. The PCN‐PPy variants‐based MOFtors, combining the advantages of those two components, are anticipated to exhibit robust full‐spectrum absorption and exceptional motion performance.

**Figure 3:**
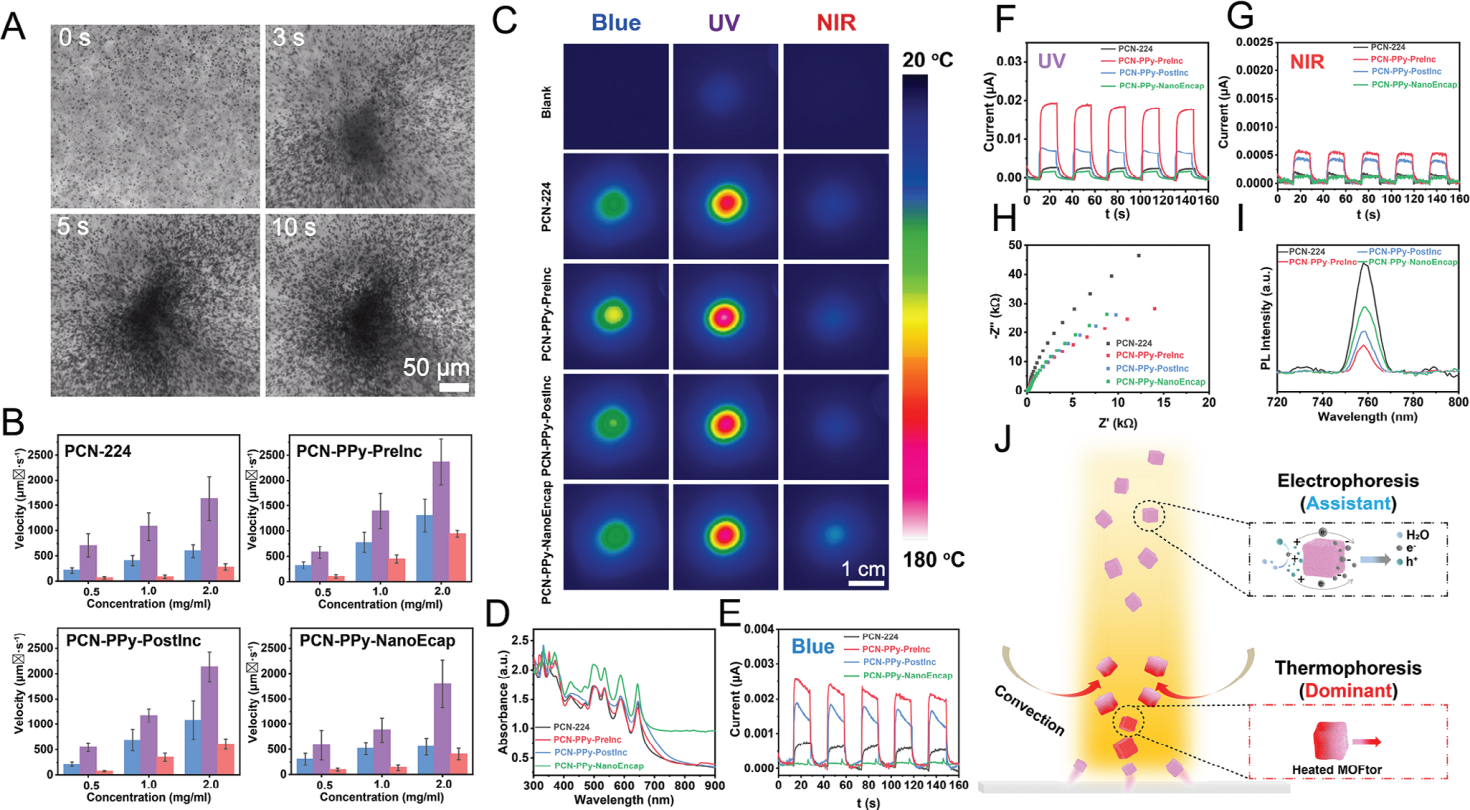
Motion and mechanism analysis. A) Time‐lapse images illustrating the formation and differences in aggregation between the PCN‐224 and PCN‐PPy variants based MOFtors before and after 1.16 W cm^−2^ of blue light irradiation. B) Velocities, C) thermal imaging, and E–G) transient photocurrent‐time curves of PCN‐224 and PCN‐PPy variants based MOFtors under different power of blue, UV, and NIR light irradiation, D) UV–vis DRS spectra of PCN‐224 and PCN‐PPy variants based MOFtors, H) electrochemical impedance spectra (EIS), I) photoluminescence spectra of PCN‐224 and PCN‐PPy variants based MOFtors. J) Scheme illustrating the motion mechanisms of MOFtors under light.

The motion behavior of these MOFtors was evaluated in water without adding any chemical fuel (such as hydrogen peroxide) under varying conditions – different concentrations of MOFtors and incident light of different wavelengths (470, 385, and 808 nm). Time‐lapse images in **Figure** **3**A demonstrated a swarming motion behavior in all MOFtors upon light illumination, gathering toward the center of the light spot and then moving convectively from the non‐irradiated area to the illuminated area. Figure 3B and Videos S1–S4 (Supporting Information) showed the average velocities of MOFtors under different concentrations and wavelengths. Regardless of the light wavelength, the velocity was proportional to the MOFtors concentration. PCN‐PPy‐PreInc exhibited the most significant velocity increase when the concentration was increased from 0.5 to 2 mg ml^−1^. The increase in the number of light‐driven micromotors amplifies heat production, stemming from the collective effect of each micromotor transforming light into thermal energy. Consequently, the overall temperature within the system rises, speeding up the development of a temperature gradient. The more rapidly established and stronger temperature gradient leads to a more distinct collective migration away from the light source, which is in agreement with previously reported works.^[^ ^15^, ^36^ ^]^ Among the various light wavelengths, MOFtors showed the fastest velocities under UV light (385 nm), with the PCN‐PPy‐PreInc based MOFtors reaching the maximum motion velocity of 2357 ± 453 µm s^−1^. Similarly, the PCN‐PPy‐PreInc‐based MOFtors also demonstrated superior velocities relative to other variants‐based MOFtors under identical experimental conditions. Then, we assessed the motion stability of PCN‐PPy‐PreInc‐based MOFtors in media with different pH values. The results indicated that MOFtors exhibited excellent motion stability at a wide range of pH, namely, 0, 3, 9, and 12 (Figure S8, Supporting Information). These distinct advantages are attributed to their unique light‐responsive properties and the underlying motion mechanism, inferred to be intricately linked to the combined light‐absorbing, photocatalytic, and photothermal properties of both PCN‐224 and PCN‐PPy variants.

To exclude the movement of micromotors caused by uneven light convection in the solution itself, polystyrene (PS) microspheres of the same concentration and similar size were used as a control group. In Video S5 (Supporting Information), it was found that the PS microspheres only displayed Brownian motion in the same environment and under the same lighting conditions. Thus, the influence of the non‐uniform light convection of the solution itself can be ruled out. Then, Infrared thermography images in Figure 3C, captured post‐irradiation with light of different wavelengths (470, 385, and 820 nm) for one minute, revealed higher temperatures for PCN‐PPy variants compared with PCN‐224. It confirms that PPy doping could enhance the photothermal ability. Furthermore, wavelength‐dependent temperature changes were detected, where PCN‐PPy‐PreInc showed a larger temperature increase under blue and UV light, while PCN‐PPy‐NanoEncap exhibited a more pronounced increase under NIR light irradiation, suggesting that PPy incorporation in PCN‐PPy‐NanoEncap maximizes the photothermal effect in the range of NIR light, while PCN‐PPy‐PreInc was more efficient under blue light. Specific temperature values were shown in Table S3 (Supporting Information). PCN‐PPy‐PreInc displayed high velocities in blue and UV light. In contrast, PCN‐PPy‐NanoEncap showed lower velocities under NIR light. These differences may be attributed to the large loading of PPy NPs, which increases the overall weight of MOFtors. To demonstrate their weight differences, uniform MOFtors aqueous dispersions of PCN‐224 and PCN‐PPy variants were investigated, and their sedimentation was monitored. As shown in Figure S9 (Supporting Information) (settling for 1 h), the sinking speed of PCN‐PPy‐NanoEncap was the fastest, indicating a heavier weight compared with the other MOFtors. Besides, the inherent hydrophobicity of PPy plays a crucial role in the functionality of MOFtors based on PCN‐PPy variants.^[^ ^15c^ ^]^ Contact angle tests (Figure S10, Supporting Information) revealed that PPy is hydrophobic, whereas PCN‐224 is hydrophilic. Through in situ polymerization of PPy in PCN‐PPy‐PreInc, the channels are less prone to wetting by water, thereby facilitating easier movement. The UV–vis diffuse reflectance spectroscopy (DRS) of PCN‐224 and PCN‐PPy variants in Figure 3D display strong light absorption peaks, i.e., the Soret band ≈400 nm and the Q‐band at ≈550 nm, attributed to their organic ligand TCPP.^[^ ^37^ ^]^

In addition to the photothermal contribution to the motion behavior of MOFtors, the influence of photoelectrophoresis was also thoroughly assessed. First, we assessed the photogenerated electronic capacity of MOFtors by cyclic voltammetry (CV) in a three‐electrode configuration, as shown in Figure S11 (Supporting Information) (with detailed methodology provided in Supporting Information). Upon irradiation with various wavelengths of light, including single‐wavelength light (470 nm blue, 385 nm ultraviolet light, and 820 nm near‐infrared light), both PCN‐224 and PCN‐PPy variants showed a higher current response compared with their performance in the dark. This indicates an enhanced charge generation capability and separation efficiency upon illumination. Second, in the instantaneous photocurrent response (*I*–*t*) experiments, all I‐t curves displayed significant and repeatable responses under blue (470 nm), ultraviolet (385 nm), or near‐infrared light (820 nm). Among these, PCN‐PPy‐PreInc displayed the highest current densities (Figure 3E–G) with values of 0.00255 and 0.00056 µA cm^−2^ under blue light and NIR light, respectively, which were 3 times higher than that of PCN‐224. Additionally, under UV light, the current density increased to 0.0193 µA cm^−2^, 9 times higher than that of PCN‐224. These results indicated that PCN‐PPy‐PreInc possesses superior charge generation ability and charge separation efficiency. Electrochemical impedance spectroscopy (EIS) analysis (Figure 3H) revealed that PCN‐PPy‐PreInc had a smaller impedance spectrum radius compared with the original PCN‐224, indicative of the smallest electron transfer resistance. This implied that the PPy incorporation can reduce interface transfer resistance, thereby enhancing photogenerated charge separation and transfer. EIS patterns under blue (470 nm), UV (385 nm), and NIR light (820 nm) irradiation are detailed in Figure S12 (Supporting Information), with the same pattern as those in dark conditions. Finally, photoluminescence spectra (PL) analyses in Figure 3I showed a reduction in PL intensities for all PCN‐PPy variants compared with PCN‐224, where PCN‐PPy‐PreInc showed the most significant decrease, suggesting a strong suppression of electron recombination in PCN‐PPy‐PreInc. This suggested that photogenerated electrons may influence MOFtors movement through the inducing self‐electrophoresis, generated by ions gradients. The impact of photoelectrophoresis on the movement of MOFtors was investigated in a NaCl electrolyte solution. A slight decrease in the velocity of MOFtors within the NaCl solution was observed, suggesting the presence of photoelectrophoresis in the motion system. However, this effect did not appear to be the predominant factor influencing their mobility (Figure S13 and Video S6, Supporting Information). This finding aligns with our previous results, indicating a consistent behavior pattern under similar conditions.^[^ ^25^ ^]^

Therefore, the motion mechanism of MOFtors can be explained by the contributions of two primary sources (Figure 3J). First, the photothermal effect plays a significant role. When the MOFtors are exposed to light, they undergo asymmetric heating, which produces a thermal gradient between the irradiated and non‐irradiated areas, leading to the natural convection of water and generating a fluid resistance that propels the MOFtors from the periphery of the light source to its center. The second contribution to motion is triggered by photoelectrophoresis, which is driven by photogenerated electrons. Under illumination, these electrons create a concentration gradient that forms a local electric field, inducing an electrophoresis force and facilitating the movement of MOFtors.^[^ ^25^, ^38^ ^]^ Collectively, the intricate interplay of the photothermal effect and photoelectrophoresis orchestrates the motion of MOFtors when exposed to light.

### 2.3 Catalytic Performance of PCN‐224 and PCN‐PPy Variants Based MOFtors

#### 2.3.1 TCH Removal Capacities and Stabilities

MNMs have been widely used in environmental remediation in recent years because of their functional versatility, transport capacity, and enhanced solute exchange capability. Meanwhile, MOFtors, combining the features of MOFs and MNMs, are emerging as promising candidates with significant potential across diverse research domains, particularly in environmental remediation of pollutants, including difficult‐to‐degrade drugs and therapeutic substances. TCH, for example, is one of the most widely used broad‐spectrum antibiotics in livestock production and clinics because of its low price. However, its stable structure comprising four aromatic rings challenges its mineralization and is often detected in water, posing serious harm to human and ecological health. In this work, TCH is taken as a typical antibiotic pollutant to study the degradation performances of MOFtors.

In our experiments, we utilized a xenon lamp (300 W, 400–1200 nm) as the light source to closely replicate natural sunlight conditions, which is a common practice in most degradation studies.^[^ ^39^ ^]^ The results of infrared thermal imaging images, photocurrent, and impedance tests under xenon lamp irradiation for various samples were presented in Figure S14 and Table S3 (Supporting Information). These results exhibited patterns consistent with those observed under specific wavelengths of light irradiation, indicating that the motion behavior of the samples under xenon lamp illumination was similar to that observed under specific wavelengths of light. It was worth mentioning that the light in the wavelength range of visible light (400–750 nm) not only triggered the motion of MOFtors together with the light of near‐infrared wavelength (750–1200 nm), but also made a significant contribution to its photocatalytic activity. In order to further prove our conjecture, the macroscopic motion of MOFtors in Video S7 (Supporting Information) was photographed and found to be highly consistent with the microscopic motion under specific wavelengths of light. This consistency further suggested that the motion mechanism involved a combination of both photothermal effect and photoelectrophoresis. The integration of PMS into the catalytic system significantly augments the synergy between MOFtors and SR‐AOPs, thereby effectively boosting the catalytic level. However, the addition of PMS introduced a high concentration of electrolytes into the system, which could potentially influence the motility of MOFtors. To address this, the motion velocities of MOFtors in PMS solution were analyzed in Figure S15 and Video S8 (Supporting Information). It is found that irrespective of the light source, MOFtors still maintained robust motility levels in the PMS solution. Strikingly, under blue light illumination, PCN‐PPy‐PreInc can reach a velocity of 962 ± 200 µm s^−1^. This robust movement capability underlined the suitability of MOFtors for TCH degradation experiments, ensuring their effective application even in the presence of high electrolyte concentrations due to PMS.

**Figure 4:**
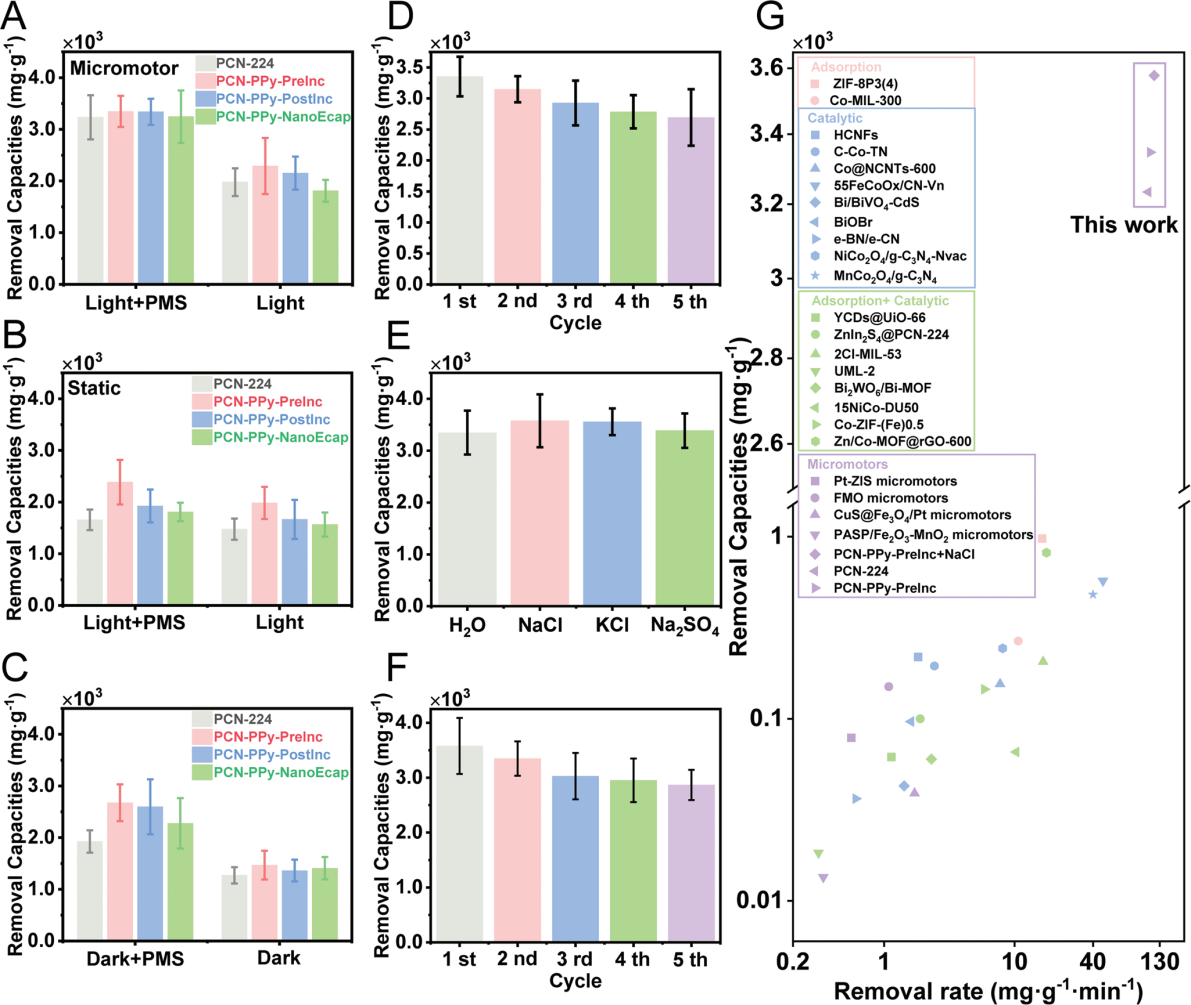
Analysis of TCH removal. A–C) Removal capabilities of TCH under different conditions, D) TCH removal capabilities for five consecutive cycles of PCN‐PPy‐PreInc under Xenon lamp irradiation in DI aqueous solution, E) TCH removal capabilities of PCN‐PPy‐PreInc based MOFtors in different salt solutions (1 m NaCl, Na_2_SO_4_ and KCl), F) TCH removal capabilities for five consecutive cycles of PCN‐PPy‐PreInc under Xenon lamp irradiation in 1 m NaCl solution, G) performance comparison with other literature.

Various control experiments were carried out to explore its effect on the catalytic removal of TCH coupled with the PMS activation approach. The line graphs of removal performance over time in relation to the removal of TCH were shown in Figure S16 (Supporting Information). The removal performances of TCH under various conditions were shown in **Figure** **4**A–C to determine the effects of different conditions (light, PMS, and motion) on the removal of TCH. PCN‐224 and PCN‐PPy variants demonstrated differing removal efficiencies when exposed to light, both in a dynamic state (free‐moving MOFtors) and a static condition (where the MOFtors were intentionally immobilized at the bottom, as detailed described in the Supporting Information). The TCH removal efficiency of MOFtors outperformed that achieved solely under xenon lamp irradiation, reaching a maximum efficiency of 2291 ± 543 mg g^−1^, which is ≈1.5 times that without motion. The enhanced TCH removal efficiency was attributed to the strong 3D motion character of MOFtors. Under light irradiation, the removal performance of motile PCN‐PPy‐PreInc increased by 1.2‐fold in 30 min compared with PCN‐224. The addition of PMS enabled PCN‐PPy‐PreInc a better removal performance, reaching up to 3347 ± 302 mg g^−1^, which was 1.5 times higher than that achieved under light irradiation only. These results indicated that PCN‐PPy‐PreInc intrinsically displayed excellent adsorption and removal performance. The incorporation of PMS into the system could effectively trigger the generation of active SO_4_^•−^ radicals. In contrast, under the same light exposure but in a static state, the removal efficiencies of PCN‐PPy variants were all reduced, illustrating the importance of the motion on catalytic performance. Removal performance in the dark condition was evaluated in Figure 4C, where the adsorption efficiency of the MOFs alone and their partial activation by the addition of PMS were examined. As a comparison, without adding samples (PCN‐224 or PCN‐PPy variants), PMS will only partially degrade TCH regardless of the presence or absence of visible light, indicating that the trace amounts of active free radicals generated in this way are insufficient to efficiently remove TCH without an activator. At the same time, experiments conducted in the presence of only light showed that the removal of TCH was negligible (Figure S17, Supporting Information). Therefore, prior to the practical TCH removal experiment (light illumination and PMS incorporation), pre‐treatment in the dark to achieve adsorption–desorption equilibrium was performed. In our work, the as‐prepared MOFtors based on PCN‐PPy‐PreInc demonstrated exceptional catalytic performance in removing the antibiotic TCH. Their effectiveness significantly surpassed that of most conventional photocatalysts, both in terms of the removal capacity and rate (see Figure 4G; and Table S4, Supporting Information for detailed comparison). This outstanding achievement underscores the advanced efficiency of PCN‐PPy‐PreInc based MOFtors, positioning them as a superior solution for tackling TCH contamination.

In terms of practical use, the stability and recyclability of the prepared MOFtors based on PCN‐PPy‐PreInc were carried out and shown in Figure 4D. The removal efficency of MOFtors maintains 80.5% after five consecutive photocatalytic reactions. Considering the inevitable loss of photocatalysts during the recycling process, the removal efficiency was maintained at a fairly high level. Furthermore, comprehensive statistical analysis was conducted on the movement behavior of PCN‐PPy‐PreInc post‐30 min Xenon lamp irradiation during the degradation experiment. It was noted that the velocity of PCN‐PPy‐PreInc under different light conditions remained consistently similar to its pre‐irradiation state. This finding underscored the resilience of the PCN‐PPy‐PreInc's motion behavior, indicating that its motion capability was well‐preserved even after exposure to light irradiation (Figure S18 and Video S9, Supporting Information). To further visualize the microstructure stability of MOFtors, five time‐used MOFtors based on PCN‐PPy‐PreInc were characterized by SEM and XRD. As shown in Figure S19 (Supporting Information), the MOFtors maintained a morphology similar to that of the fresh samples. In addition, the characteristic diffraction peaks of the used MOFtors were consistent with the fresh samples, and no additional diffraction peaks were observed, suggesting good physical and chemical stability.

In real‐world applications, wastewater containing antibiotic TCH often has a high salt concentration.^[^ ^40^ ^]^ To address this, the effect of electrolytes on the TCH degradation in saline wastewater has been studied. Three different salts, i.e., NaCl, KCl, and Na_2_SO_4_, at an equal concentration (1 m), respectively, were added to the TCH solutions to simulate the practical environmental conditions. For these experiments, we selected PCN‐PPy‐PreInc based MOFtors, which exhibited the best motion and degradation performance in a salt‐free environment, to investigate their motion capability and photocatalytic activity in a PMS/Light system.

Focusing first on the NaCl solution, the motion behavior of PCN‐PPy‐PreInc‐based MOFtors under blue, UV, and NIR light was studied (Figure S13 and Video S6, Supporting Information). Remarkably, the velocities of these MOFtors remained high and did not show significant reduction under any type of light. Upon adding PMS, the velocities still maintained high levels, reaching 639 ± 92, 1575 ± 221, and 449 ± 44 µm s^−1^ under blue, UV, and NIR light, respectively (Figure S20 and Video S10, Supporting Information). This sustained high‐velocity performance under saline conditions is highly advantageous for their application in degradation experiments, demonstrating the robustness and effectiveness of PCN‐PPy‐PreInc based MOFtors in challenging saline environments. As shown in Figure 4E, the TCH removal performances with NaCl, KCl, and Na_2_SO_4_ were estimated to be 3578 ± 510, 3558 ± 313, and 3388 ± 423 mg g^−1^, respectively. Compared with the removal performance in the deionized water system, the TCH degradation performance with chloride ions was further improved due to the generation of chlorine free radicals (Cl•). Because of its strong oxidizing properties, Cl• is very effective in degrading organic pollutants.^[^ ^41^ ^]^

Figure 4F showed the cycling stability of MOFtors based on PCN‐PPy‐PreInc in the TCH solution containing NaCl. Note that after five cycles, the MOFtors still preserved a degradation efficiency of 80.2%. Besides, the sample exhibited remarkable stability in both morphology and crystal structure (Figure S21, Supporting Information). Even after a one‐month storage period at room temperature, the MOFtors did not exhibit noticeable alterations in their morphological structure or motion behavior. This observation highlights the exceptional long‐term stability of the MOFtors, demonstrating their resilience and consistent performance over time (Figures S22 and S23 and Video S11, Supporting Information). This structural integrity over time underscored the robustness of the material, a crucial factor for practical applications.

#### 2.3.2 Removal Mechanism of PCN‐224 and PCN‐PPy Variants

**Figure 5:**
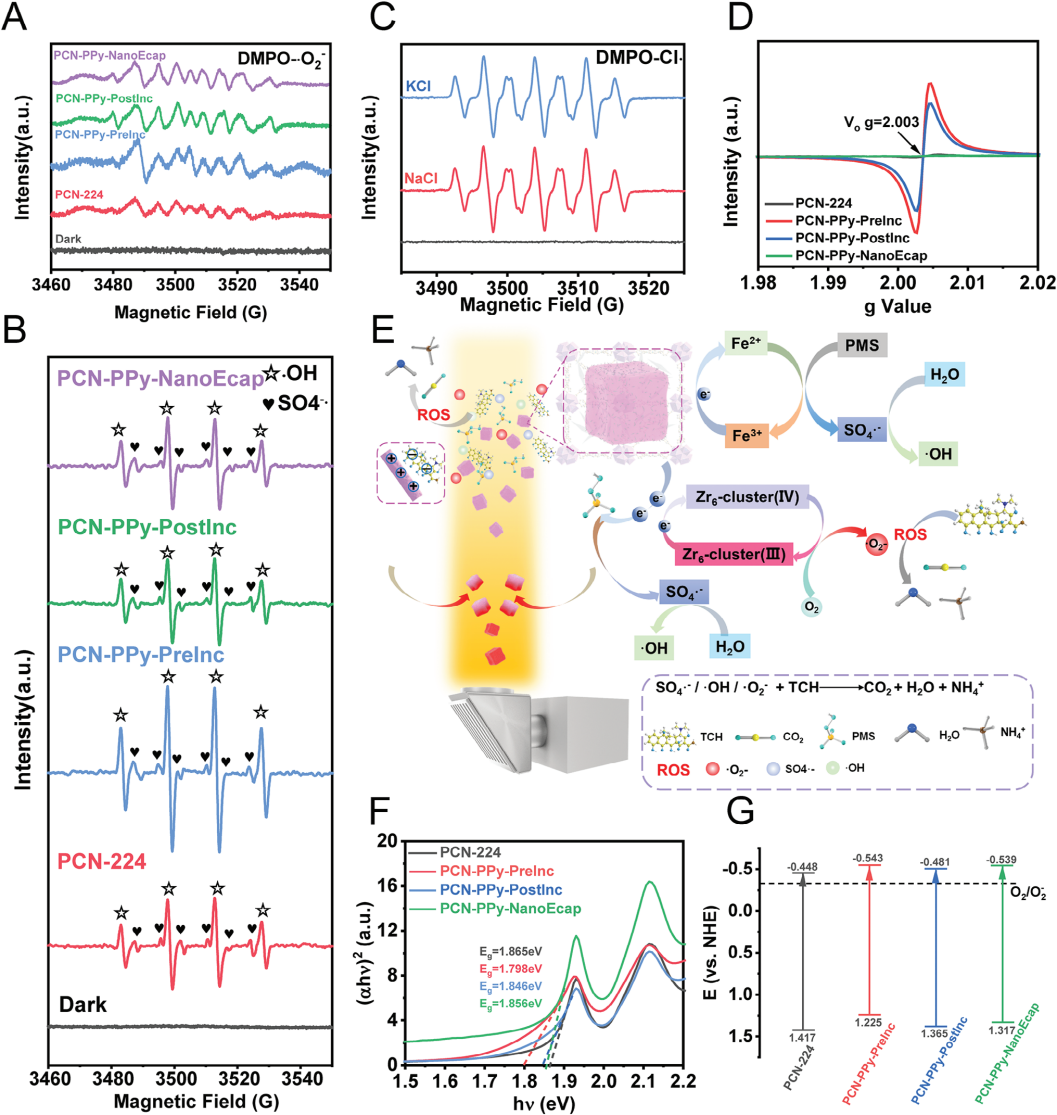
Analysis of the TCH degradation mechanism. A,B) EPR spectra of •O_2_^−^, •OH, and SO_4_^•−^ of PCN‐224 and PCN‐PPy variants based MOFtors after Xenon Lamp irradiation, C) EPR spectra of Cl• of PCN‐PPy‐PreInc based MOFtors in 1 m NaCl and KCl, D) EPR spectra with the g values for PCN‐224 and PCN‐PPy variants based MOFtors, E) Possible mechanism of PMS activation for TCH degradation, F) Plots of the transformed Kubelka‐Munk function versus the energy of light and G) Energy band structure and bandgap diagram of PCN‐224 and PCN‐PPy variants based MOFtors.

The active species produced during the photocatalytic reaction are crucial for the removal of TCH. Electron paramagnetic resonance spectroscopy (EPR) was performed during the TCH removal process to study the photodegradation reaction mechanism in detail. The reactive oxygen species (ROS) produced in NaCl solution under light irradiation was characterized in **Figure** **5**A–C. The absence of peaks representing •OH and •O_2_^−^ in the EPR spectra under dark conditions without the addition of PMS indicated that •OH and •O_2_^−^ cannot be produced only under dark conditions. In contrast, characteristic peaks of •O_2_^−^ were detected for different PCN‐PPy variants under light exposure, and PCN‐PPy‐PreInc, with the best TCH removal performance, presented the strongest •O_2_^−^ signal. Meanwhile, the EPR results in Figure 5C indicated the generation of Cl· under light irradiation. With the addition of PMS, both •OH and SO_4_^•−^ were detected for PCN‐224 and PCN‐PPy variants after light exposure, and the PCN‐PPy‐PreInc exhibited the highest intensity, which was in good agreement with the actual removal performances, and different degrees of reduction of •O_2_^−^ peak intensity were also detected (Figure S24A, Supporting Information). Besides, as shown in Figure S24B (Supporting Information), •OH cannot be produced by light irradiation alone without PMS. In addition, as shown in Figure 5D, oxygen vacancies were detected in both PCN‐224 and PCN‐PPy variants, with PCN‐PPy‐PreInc displaying the highest density of oxygen vacancies. Finally, the surface charges of different samples were determined using Zeta potential analysis to assess the synergistic influence of surface charge on the functional performance of the MOFtors. Incorporating PPy into PCN‐224 frameworks resulted in a change from negative to positive surface charge. Since TCH is a negatively charged compound, the electrostatic attraction between TCH and PPy‐modified PCN‐224 facilitated a closer interaction (Figure S25, Supporting Information),^[^ ^42^ ^]^ thus promoting the effective adsorption and subsequent degradation of TCH.

Based on the above experimental results and previous studies, a possible mechanism for TCH removal in MOFtors/PMS system was proposed (Figure 5E). First of all, the porphyrin in the MOFtors can transition to an excited state upon light exposure. Subsequently, the high valence Zr(IV) received photogenerated electrons from the ligand, becoming a lower valence Zr(III) cluster. Owing to the strong reducing power of Zr (III), the dissolved O_2_ in water can then be reduced to •O_2_^−^. PMS can be activated by trapping photogenerated electrons to produce the active radicals of SO_4_^•−^, which react with H_2_O to form •OH. Iron ions in PCN‐PPy‐PreInc and PCN‐PPy‐PostInc also participated in the reaction, wherein photogenerated electrons can also be transferred from the ligands to the Fe^3+^ centers to form Fe^2+^, which subsequently engaged in the activation of PMS, ultimately leading to the generation of more SO_4_^•−^.

Accurate bandgap energy (Eg) values were calculated to further gain insights into the photocatalytic mechanism, shedding light on the generation and transfer of photogenerated carriers in the catalyst during the TCH removal process. From the UV–vis DRS results, a Tauc plot (Figure 5F) was constructed using the Kubelka‐Munk function. The results revealed that PCN‐224 presented a direct bandgap (Eg) of 1.865 eV. Notably, the incorporation of PPy led to a reduction in the bandgap, with PCN‐PPy‐PreInc displaying the narrowest gap. As shown in Figure S26 (Supporting Information), the Mott‐Schottky (MS) curve for PCN‐224 showed a linear relationship with a positive slope, characterizing it as a typical n‐type semiconductor. The flat band potential (E_FB_) for PCN‐224 and the three kinds of PCN‐PPy were determined to be −0.648, −0.743, −0.681, and −0.731 V, respectively (relative to Ag/AgCl reference electrode). According to the empirical formula Eg = E_VB_(valence band) – E_CB_(conduction band),^[^ ^43^ ^]^ the potentials of E_CB_ and E_VB_(vs NHE) are shown in Figure 5G. Note that the E_CB_ of these catalysts were all more negative than the redox potential of O_2_/•O_2_^−^ (−0.33 V vs NHE), indicating a higher reducing ability of the catalysts. These results strongly suggested that in situ polymerization of PPy within the MOFs pores leaded to a higher catalytic activity of the MOFtors in comparison to the PPy directly encapsulated.

Furthermore, the light‐driven movement of MOFtors played a crucial role in enhancing the photocatalytic process. This motion facilitated solute exchange and enhanced surface charge effects, thus improving the interaction between reactive oxygen species (ROS) and the target contaminant, TCH. ROS such as SO_4_^•−^, •OH, and •O_2_^−^ are highly reactive but short‐lived, requiring close proximity to effectively degrade TCH. The movement of MOFtors helped to extend the effective range of these ROS and improved the exposure of TCH to the reactive sites on the MOFtors' surface by promoting better mixing and interaction in the solution. In conclusion, the coordinated action of MOFtor motions, surface charges, reactive ROS, and the MOFtors themselves synergistically contributed to the highly efficient degradation of TCH during the photocatalytic process.

#### 2.3.3 Possible Removal Pathways of TCH

**Figure 6:**
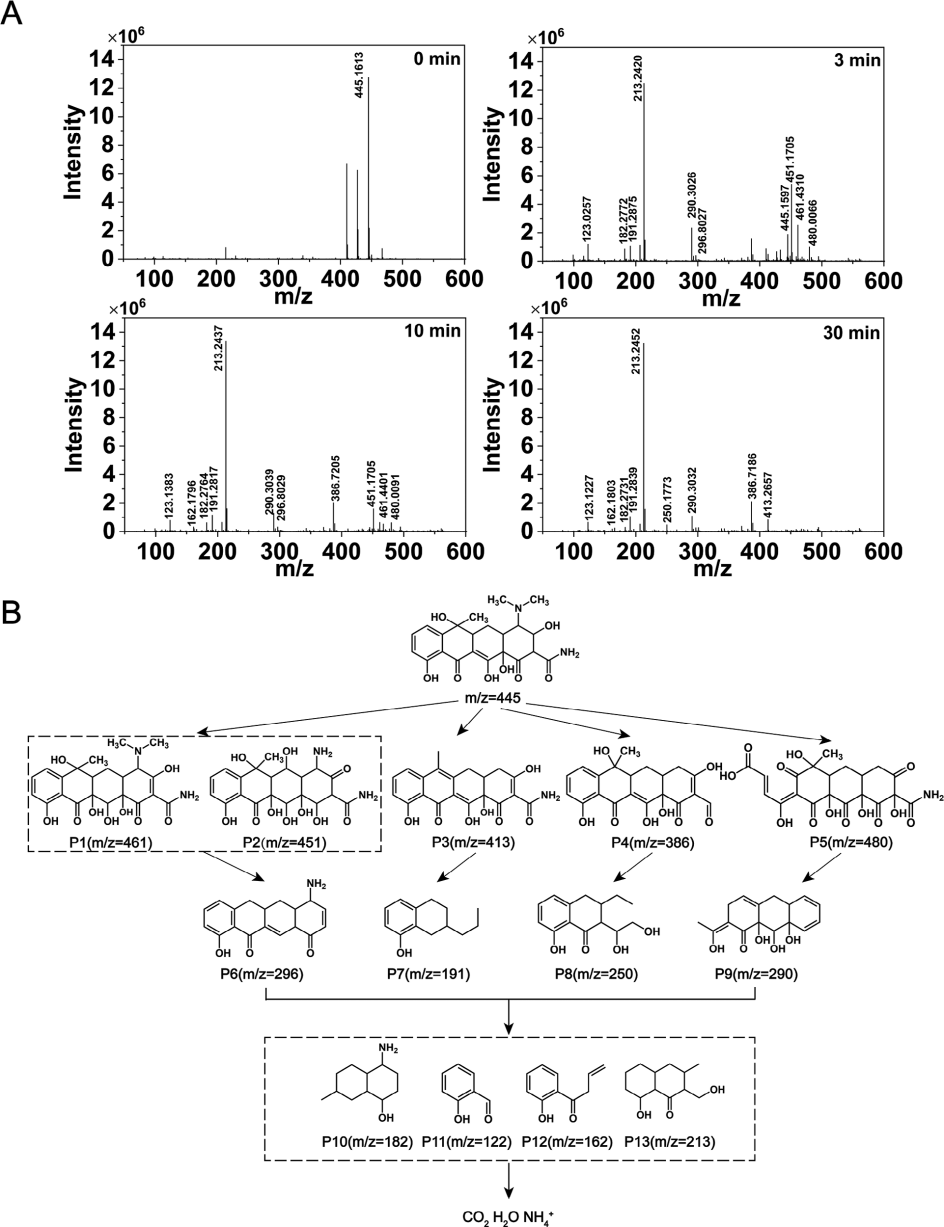
Analysis of intermediates. A) Mass spectra of photocatalytic degradation of TCH and its related intermediates at different times. B) Possible degradation pathway of TCH.

To elucidate the intermediates involved in the TCH removal process, the degradation of TCH in the MOFtors/PMS/Light system over various time intervals was analyzed using high‐performance liquid chromatography‐time‐of‐flight (HPLC‐TOF) spectrometry. In the beginning, distinct TCH peaks were evident. However, as the degradation process progressed, there was a notable decrease in the intensity of the TCH peaks, indicative of the rapid decline in the TCH concentration. Several major intermediates were identified, and their corresponding spectra and structures, along with possible products, were shown in **Figure** **6**A and Table S5 (Supporting Information). Key functional groups in TCH, such as hydroxyls, dimethylamino groups, ketones, and formyls, are likely oxidized to form less toxicity intermediates.^[^ ^44^ ^]^ Four potential TCH degradation pathways, each leading to low molecular weight products, were proposed in Figure 6B. In pathway I, the C═C double bond in TCH was attacked by •OH, resulting in the formation of hydroxyl and ketone groups and yielding products P1 (m/z 461) and P2 (m/z = 451), the latter through additional hydroxyl oxidation and dehydration.^[^ ^45^ ^]^ Pathway II involved the loss of the dimethylamino group, likely due to the low bond energy in the C─N linkage during dehydration, producing P3 (m/z = 413).^[^ ^46^ ^]^ In pathway III, TCH molecules were attacked by SO_4_^•−^ and •OH, leading to deamination and dealkylation, with the formation of product P4 (m/z = 386).^[^ ^47^ ^]^ Pathway IV indicated the removal of the dimethylamino group by O_2_^−^· attack, with the addition of another ketone group and carboxyl group, forming intermediate P5 (m/z = 480).^[^ ^48^ ^]^ Subsequent oxidation of these intermediates leaded to the products P6‐P9 (m/z = 296, 190, 250, and 290), which were associated with ring‐opening reactions, carbon bond cleavage, demethylation, and dihydroxylation.^[^ ^49^ ^]^ These compounds were then subject to decarbonization, dehydration, and ring opening, transforming into low weight organic compounds such as P10‐13, and eventually mineralizing into inorganic substances such as CO_2_, H_2_O, and ammonium through further oxidation.

## 3 Conclusion

In summary, we have successfully developed 3D motile MOFtors that operate under ultraviolet, visible, and near‐infrared light, harnessing the synergistic effect of thermophoresis and electrophoresis. The motion behavior and photocatalytic properties of these MOFtors (PCN‐PPy) were finely adjusted by incorporating the photoactive component PPy using three different approaches. Strikingly, the MOFtors based on PCN‐PPy‐PreInc achieved a remarkable maximum velocity of 1305 ± 327 µm s^−1^. The robust 3D motion of these MOFtors significantly enhances fluid mixing, thereby expanding the range and effectiveness of short‐lived radicals. This feature, along with their high ion tolerance, distinguished them from traditional light‐driven micromotors, particularly in highly saline solutions. These MOFtors not only maintained high movement performance but also exhibited superior removal capacity. Furthermore, the addition of PMS to the system resulted in an exceptional degradation efficiency of 3347 ± 302 mg g^−1^, a significant advancement over current technologies when combined with SR‐AOPs. Additionally, the presence of chlorine ions in the solution was found to further boost the TCH removal (3578 ± 510 mg g^−1^) because of the activation of chlorine‐free radicals. This study emphasized the practicality of MOFtors as a cost‐effective solution for water treatment, offering recyclability without the risk of secondary contamination and promising advancements in efficient water treatment technologies.

## Conflict of Interest

The authors declare no conflict of interest.

## Supporting information

Supporting Information

Supplemental Video 1

Supplemental Video 2

Supplemental Video 3

Supplemental Video 4

Supplemental Video 5

Supplemental Video 6

Supplemental Video 7

Supplemental Video 8

Supplemental Video 9

Supplemental Video 10

Supplemental Video 11

## Data Availability

The data that support the findings of this study are available in the supplementary material of this article.
