# Supplementary material for: Revolutionizing Tetracycline Hydrochloride Remediation: 3D Motile Light‐Driven MOFs Based Micromotors in Harsh Saline Environments
Source: Adv Sci (Weinh). 2024 Aug 29;11(40):2406381. doi: 10.1002/advs.202406381 (PMC11516158; doi:10.1002/advs.202406381)
Supplement: Supplementary file 1 — Supporting Information [file ADVS-11-2406381-s001.docx]

Supporting Information

**Revolutionizing Tetracycline Hydrochloride Remediation: 3D Motile Light-Driven MOFs Based Micromotors in Harsh Saline Environments**

Yu Zhao, ^[a]^ Jiawei Lin, ^[a]^ Qing Wu, ^[a]^ Yulong Ying, *^[a]^ Josep Puigmartí-Luis,*^[b], [c]^ Salvador Pané,*^[d]^ Sheng Wang*^[a]^

^[a]^ Yu Zhao, Jiawei Lin, Qing Wu, Yulong Ying*, and Sheng Wang*^[a]^

School of Materials Science and Engineering

Zhejiang Sci-Tech University

Hangzhou, 310018, P. R. China

E-mail: yingyulong@zstu.edu.cn (Yulong Ying)

^[b]^ Departament de Ciència dels Materials i Química Física, Institut de Química Teòrica i Computacional, University of Barcelona, Martí i Franquès, 1, 08028 Barcelona, Spain

^[c]^ Institució Catalana de Recerca i Estudis Avançats (ICREA), Pg. Lluís Companys 23, 08010 Barcelona, Spain

^[d]^ Multi-Scale Robotics Lab, Institute of Robotics and Intelligent Systems, ETH Zurich, Tannenstrasse 3, 8092 Zurich, Switzerland.

## Table of Contents

[Table of Contents 2](#_Toc161261322)

[Supplementary Experimental Sections 3](#_Toc161261323)

[Materials. 3](#_Toc161261324)

[Characterization. 3](#_Toc161261325)

[Synthesis of PCN-224. 4](#_Toc161261326)

[Synthesis of PCN-PPy-PreInc. 4](#_Toc161261327)

[Synthesis of PCN-PPy-PostInc. 4](#_Toc161261328)

[Synthesis of PCN-PPy-NanoEcap. 4](#_Toc161261329)

[Synthesis of PPy. 4](#_Toc161261330)

[Motion of the PCN-224 and PCN-PPy variants based MOFtors under light. 5](#_Toc161261331)

[Photoelectrochemical measurements. 6](#_Toc161261332)

[Photothermal measurements. 6](#_Toc161261333)

[Photocatalytic experiments of the PCN-224 and PCN-PPy variants based MOFtors. 6](#_Toc161261334)

[Supplementary Figures 8](#_Toc161261335)

[Supplementary Tables 18](#_Toc161261336)

[References 21](#_Toc161261337)

## Supplementary Experimental Sections

Materials. Zirconyl chloride octahydrate (ZrOCl_2_⸳8H_2_O, 99.9%), 4,4,4,4-(Porphine-5,10,15,20-tetrayl)tetrakis(benzoic acid) (H_2_TCPP, 97%), peroxymonosulfate (PMS, 4.5 % active oxygen), iron(III) chloride (FeCl_3_, AR, 99%), polyvinyl Alcohol (PVA, Mw: 31000), and pyrrole monomer (98%) were purchased from Shanghai Macklin Reagent Co., Ltd. Acetic acid (CH_3_COOH, ≥99.5%), N,N-dimethylformamide (DMF, ≥99.5%), methyl alcohol (CH_3_OH, ≥99.9%), and ethanol (CH_3_CH_2_OH, ≥99.9%) were obtained from Hangzhou Shuanglin Chemical Reagent Co., Ltd. Sodium chloride (NaCl, AR), potassium chloride (KCl), and sodium sulfate (Na_2_SO_4_) were purchased from Hangzhou Gaojing Fine Chemical Industry Co., Ltd. All chemicals were procured from commercial suppliers and were used as received without further purification, except where specifically noted. Milli-Q water was used in all experiments (Millipore, 18.2 MΩ.cm).

Characterization. SEM images were acquired with a field emission scanning electron microscope (FESEM, Hitachi S-4800, Japan). Energy-dispersive X-ray spectroscopy (EDX) analysis data were performed using a Horiba EDX detector system integrated with the FESEM. Transmission morphology images, high-resolution TEM images, EDX mapping images, and selected area electron diffraction patterns were collected employing a FEI Tecnai transmission electron microscope (FEI, Hillsboro, OR, USA), outfitted with an Oxford instruments X-Max. The power X-ray diffraction (XRD) patterns were obtained using a Bruker Focus D8 diffractometer (Germany), equipped with a fine focus Cu sealed tube (λ=1.54178 Å), operating at 40 kV and 40 mA. Fourier transform infrared (FTIR) measurements were performed on Nicolet iS50 (USA) to investigate molecular vibrations and chemical bonds. Raman scattering spectra were collected using an inVia Reflex micro confocal Raman microscope (HORIBA, France), offering insights into molecule structures. X-ray photoelectron spectroscopy (XPS) analysis was carried out on Thermo Scientific ESCALAB Xi^+^ (USA), providing detailed information on elemental and chemical states. Nitrogen adsorption–desorption measurements were carried out using Micromeritics 3Flex instrument (USA) at 77 K to 1.0 bar. Samples were activated under vacuum at 150 °C for 8 h prior to measurement. Contact angle measurements were performed on a Dataphysics OCA 25 instrument. UV-Visible diffuse reflectance spectra (DRS) were recorded on an ultraviolet-visible spectrophotometer (UH4150, Hitachi, Japan) with an integrating sphere attachment. Steady-state photoluminescence (PL) measurements were performed using a Hitachi F-4600 instrument (Hitachi, Japan), equipped with a 440 nm laser excitation source. Zeta potential tests were carried out with a Malvern Zetasizer Nano ZSE instrument (UK) to characterize the surface charge. Electron paramagnetic resonance spectrometer (EPR) was performed on an A300-10/12 spectrometer (Bruker BioSpin Co., Karlsruhe, Germany) to capture spin signals of reactive oxygen species, with samples homogeneously dispersed in aqueous solution and exposed to Xenon lamp irradiation. In detail, the PCN-224 and PCN-PPy variants were homogeneously dispersed in an aqueous solution (0.5 mg mL^-1^) by ultrasonic treatment. Then, 50 μL of 5,5-dimethyl-1-pyrroline N-oxide (DMPO, 100 mM) was added to the solution and thoroughly mixed to capture the spin signal of superoxide radical (⸱O_2_^-^). For the detection of other radicals, DMPO was replaced with suitable capturing agents to identify hydroxyl radical (⸱OH) and sulfate radical (SO_4_^•-^) signals. Subsequently, the resulting suspension was placed on an operating platform and subjected to irradiation from a Xenon lamp (300 W, λ>400 nm) for 3 min. Finally, the suspension was collected using capillary tubes after a thorough shaking and then positioned in the test chamber of the EPR spectrometer for measurement. The structures of intermediates were detected by the high-performance liquid chromatography-time-of-flight (HPLC-TOF) spectrometry (SYNAPT-G2-S HDMS, Waters, USA) in positive electrospray ionization (ESI^+^) mode within the m/z range of 50-1000.

Synthesis of PCN-224. The preparation of PCN-224 was slightly modified according to a previously published method.^[1]^ In particular, 100 mg of ZrOCl_2_⸳8H_2_O was dissolved in 40 mL of DMF under magnetic stirring for 30 min. Then, 20 mg of TCPP was added to the aforementioned solution, followed by stirring for 10 min. Afterward, 10 mL of acetic acid was added to the mixture and stirred for another 10 min to ensure thorough mixing. Finally, the resultant solution was transferred to the Teflon-lined autoclave (100 mL) and subjected to a reaction at 65 ^o^C for 72 h. The precipitation was separated by centrifuging at 5000 rpm⸱min^-1^ and repeatedly washed with methanol several times to remove any residual impurities. The resulting dark purple powders were finally dried in a vacuum oven at 60°C for 8 h and named PCN-224.

Synthesis of PCN-PPy-PreInc. 100 mg of ZrOCl_2_⸳8H_2_O was dissolved in 40 mL of DMF and stirred magnetically for 30 min. Then, 20 mg of TCPP was added to the solution and stirred for an additional 10 min. Subsequently, 10 mL of acetic acid was added to the mixture and stirred for another 10 min. Afterward, 560 μL of pyrrole monomer was added to the mixture and stirred for 10 min to ensure thorough mixing. The resulting mixture was then transferred to a PTFE-lined autoclave (100 mL) and reacted at 65 ^o^C for 72 h. The precipitate was separated by centrifugation at 5000 rpm⸱min^-1^ and repeated washing with methanol several times to remove any residual impurities. The red intermediate product obtained was then transferred to DI water, where FeCl_3_⸱6H_2_O was added in a molar ratio of [Pyrrole]:[Fe^3+^] = 1:3. The mixture was stirred at 0 °C for 24 h in the dark to ensure the complete reaction. Finally, PCN-PPy-PreInc was obtained by the same centrifugation and drying step as previously described.

Synthesis of PCN-PPy-PostInc. The as-synthesized PCN-224 (40 mg) was dispersed in 5 mL of ethanol, followed by the addition of pyrrole (Py) (560 μl). The mixture was then maintained under N_2_ atmosphere for 24 h. Subsequently, excess Py was removed through centrifugal washing with methanol, yielding the intermediate product of PCN-Py. The polymerization procedure involved immersing PCN-Py in 30 mL of FeCl_3_ aqueous solution, with a molar ratio of [Pyrrole]:[Fe^3+^] = 1:3. The resultant product was thoroughly washed with methanol to remove any unreacted FeCl_3_ and potential oligomers that may have formed during the reaction.

Synthesis of PCN-PPy-NanoEcap. 100 mg of ZrOCl_2_⸳8H_2_O was dissolved in 40 mL of DMF, and the solution was stirred magnetically for 30 min. Then, 20 mg of TCPP was added to the aforementioned solution, followed by stirring for 10 min. Next, 10 mL of acetic acid was added to the mixture and stirred for another 10 min. The process advanced with the incorporation of 5 mg of polypyrrole nanoparticles (PPy NPs) into the solution and stirred for a future 10 min. Finally, the mixture was transferred to a Teflon-lined autoclave (100 mL) and reacted at 65 ^o^C for 72 h. The precipitate was centrifuged at 5000 rpm⸱min^-1^ and underwent several washes with methanol to remove any residual impurities, yielding PCN-PPy-NanoEcap.

Synthesis of PPy. 4.5 g of PVA was dissolved in DI water at room temperature. FeCl_3_⸱6H_2_O was then added to the aqueous PVA solution, maintaining a molar ratio of FeCl_3_ to pyrrole at 2.3. The mixture was stirred for 1 h to ensure complete dissolution and homogeneity. Subsequently, pyrrole was introduced into the aqueous PVA/FeCl_3_ solution, initiating the polymerization reaction. The as-obtained nanoparticles were separated from the dispersion by centrifugation (10000 rpm, 10 min) and washed several times with hot DI water to remove the impurities. The collected PPy nanoparticle precipitates were dried in a vacuum oven at room temperature.

Motion of the PCN-224 and PCN-PPy variants based MOFtors under light. The motion behavior of PCN-224 based micromotors was studied under an ICX41 inverted biological microscope (Sunny Optical Technology Group Co. Ltd, China), coupled with a 20x objective. For high-speed video capture, a BFS-U3-31S4M-C (FLIR, USA) was utilized, with the footage managed via SpinView software. All recordings were conducted at room temperature at a rate of 50 frames per second (fps), facilitating subsequent object-tracking analysis using the MTrack J plugin in Image J software.

**Motion behavior of MOFtors with different concentrations and compositions.** The detailed procedure involved dropping 100 μL of MOFtors suspension with different concentrations (0.5, 1.0, 2.0 mg·mL^-1^) and compositions onto a glass slide. Their motion behaviors were observed and recorded using the microscope system by applying light at different wavelengths and power densities: 470 nm blue light at a power density of 1.16 W cm^-2^, 385 nm UV light at a power density of 2.56 W·cm^-2^, and 808 nm NIR light at a power density of 2.0 W·cm^-2^. The blue and UV light were generated employing a 5 W LED using UV, and B1 fluorescent filters. The light intensity was calculated using the PM100USB power meter coupled to a S170C sensor from Thorlabs Inc. The NIR light was provided by an external 808 nm infrared diode laser (MDL-N-808-8W, Changchun New Industries Optoelectronics Tech. Co., Ltd.).

**Motion behavior of MOFtors in solutions with different pH values.** 100 μL of a suspension, in which the concentration of MOFtors was 2.0 mg⸳mL^-1^, was precisely dispensed onto a glass slide , and the pH value of the solution was adjusted to 0, 3, 9 and 12, respectively. Then, the motion behaviors of the MOFtors were observed and recorded. The microscope system, equipped with a high-speed camera, was utilized to capture the dynamic activities of the MOFtors. This was achieved by applying light at various wavelengths and power densities, as previously described.

**Motion behavior of Polystyrene microspheres.** 100 μL of Polystyrene microspheres suspension with a concentration of 2.0 mg·mL^-1^ was precisely dispensed onto a glass slide. Then, the motion behaviors of Polystyrene microspheres were observed and recorded. The microscope system, equipped with a high-speed camera, was utilized to capture the dynamic activities of Polystyrene microspheres. This was achieved by applying light at various wavelengths and power densities, as previously described.

**Motion behavior of MOFtors in electrolyte solutions (NaCl, PMS) of different concentrations.** **NaCl Solution**: 100 μL of a suspension, containing MOFtors (concentration: 2.0 mg⸳mL^-1^) and NaCl (1 M), was precisely dispensed onto a glass slide. **PMS Solution**: 100 μL of a suspension, containing MOFtors (concentration: 2.0 mg⸳mL^-1^) and PMS (1 mM), was precisely dispensed onto a glass slide. **NaCl and PMS Solution**: 100 μL of a suspension, containing MOFtors (concentration: 2.0 mg⸳mL^-1^), NaCl (1 M), and PMS (1 mM), was precisely dispensed onto a glass slide. For each setup, the motion behaviors of the MOFtors were observed and recorded. The microscope system, equipped with a high-speed camera, was utilized to capture the dynamic activities of the MOFtors. This was achieved by applying light at various wavelengths and power densities, as previously described.

**Motion behavior of MOFtors after 30 min irradiation by Xenon lamp or being stored at room temperature for one month. Post-irradiation**: 10 mL of MOFtors suspension (concentration: 2.0 mg⸳mL^-1^) was exposed to light irradiation from a Xenon lamp for 30 minutes within a beaker. Subsequently, 100 μL of the irradiated MOFtors suspension was periodically extracted at various time intervals and dispensed onto a glass slide. **Post-Storage**: A parallel procedure was conducted with MOFtors that had been stored at room temperature for one month. 100 μL of the stored MOFtors suspension was placed onto a glass slide for motion observation and tracking. In both scenarios, their motion behaviors were observed and recorded using the microscope system. The system, equipped with a high-speed camera, captured the dynamics of the MOFtors by applying light at different wavelengths and power densities, consistent with the previous settings.

Photoelectrochemical measurements. Photocurrent measurements, including photocurrent response (I-t), electrochemical impedance spectroscopy (EIS), Mott-Schottky plots, and cyclic voltammetry (CV) were conducted on a CHI 660E electrochemical workstation in a standard three-electrode cell configuration with a 0.5 M Na_2_SO_4_ (pH = 6.8) electrolyte solution. A commercial indium tin oxide (ITO) electrode (1×2 cm^2^) served as the working electrode, complemented by a platinum foil (1×1 cm^2^) as the counter electrode and a standard Ag/AgCl as the reference electrode. The working electrode was prepared as follows: 60 μL of Nafion solution (5 wt%) was mixed with 1 mL of 4:1 (v/v) water/ethanol mixture. Then 5 mg of either PCN-224 or PCN-PPy variants were added to the mixture. Ultrasonic treatment was carried out at 15 ^o^C for 30 min to ensure uniform dispersion of the sample in the solution. Following this, 300 μL of the resulting suspension was dropped on the ITO electrode (1×1 cm^2^) and dried at 60 ^o^C. The effective loading mass of the micromotors was calculated to be 1.41 mg⸳cm^-2^. During the photocurrent testing, two light sources were used: UV-LED (UVEC-4II) spotlights (Shenzhen Lamp Co., LTD.), equipped with lasers of different wavelengths (470 nm at 0.5 W⸳cm^-2^; 385 nm at 0.285 W⸳cm^-2^, and 820 nm at 0.07 W⸳cm^-2^), and a Xenon lamp (400-1200 nm at 400 mW⸳cm^-2^). Photocurrent response curves (I-t) were recorded over 5 cycles, each cycle lasting 30 s.

Photothermal measurements. Infrared thermal images were captured by a VarioCAM head 620 infrared thermal imaging camera (InfraTec, Germany) to assess the solid photothermal properties of the samples. To ensure consistency in the measurements, samples were prepared on a coverslip, each with the same thickness and area. Each sample comprised the same quantity of corresponding solid powder (50 mg). The photothermal effect was induced by a UV-LED spotlight source illuminator (UVEC-4 II) equipped with lasers of different wavelengths and power densities. Specifically, blue light (470 nm at 0.5 W⸳cm^-2^), UV light (385 nm at 0.285 W⸳cm^-2^), NIR light (820nm at 0.07 W⸳cm^-2^), and a Xenon Lamp (400-1200 nm at 400 mW ⸳cm^-2^) were employed. During the solid photothermal measurements, infrared thermal images of the samples were systematically captured every 1 s to monitor and record the temperature changes induced by the photothermal effect.

Photocatalytic experiments of the PCN-224 and PCN-PPy variants based MOFtors. Briefly, the catalyst (1.5 mg) was dispersed into TCH solution (200 mg·L^-1^, 30 mL) and placed in the dark for 5 h to achieve adsorption-desorption equilibrium. Six groups of solutions (30 mL each) were prepared to conduct the degradation experiments under various conditions. For the first group, a Xenon lamp (300 W, 400-1200 nm) was activated after adding 61.55mg PMS to the 30mL system (≈0.03mM). The second group was only irradiated by the Xenon lamp, while the third group and the fourth group were performed in the dark conditions as the control groups. To verify the effect of motor movement on its performance, the fifth and sixth groups were carried out with catalysts fixed at the bottom of the glass bottle. In detail, the 0.5 mL of ethanol solution containing 1.5 mg of catalyst was injected into a glass bottle, and then put it on a heating table at 60 °C until the ethanol was completely evaporated. It is worth noting that the catalyst fixed on the bottom of the glass bottle can be considered as monolayer because of its very low loading amount. The monolayer assembly of catalyst on the bottom of the glass bottle were confirmed by the optical microscopy. Hence, the effect of reduced surface areas on its catalytic performance can be ignored. Other conditions are the same as those in the first and second groups. The distance between Xe lamp and the reactor is 10 cm. Through the power meter (CEL-NP2000-2(10)A, CEAU-LIGHT, Beijing), the light intensity of the Xenon lamp incident on the reactor was measured as 400 mW·cm^-2^. Unless specified otherwise, all experiments were conducted without adjusting the initial pH value. At predetermined intervals, 1mL of the solutions were taken, filtered by microfiltration membrane (0.22 μm), and analyzed by UV-vis spectrum at 357 nm to determine the TCH degradation efficiency. All degradation experiments were repeated five times, and the results were averaged to ensure reliability and accuracy.

## Supplementary Figures

**
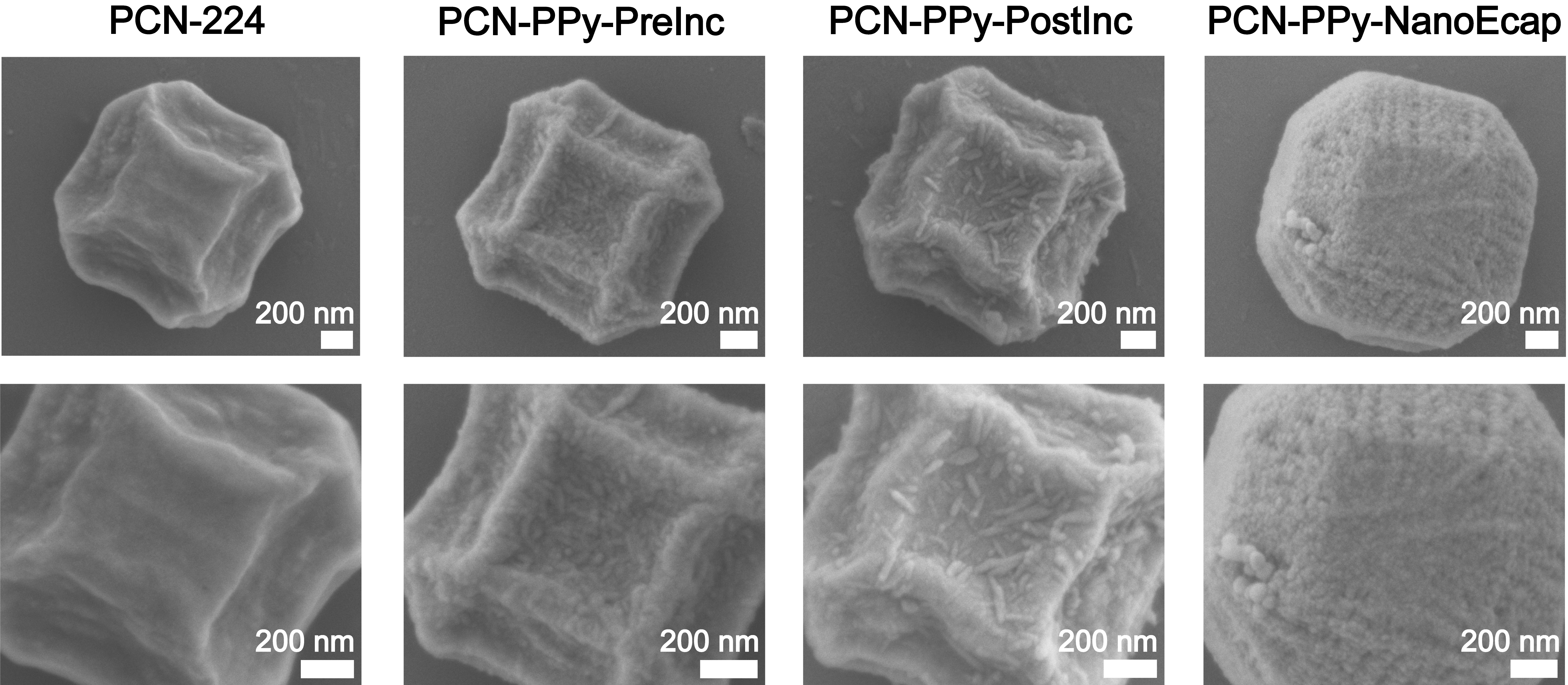
**

**Figure S1.** The high-magnification SEM images of PCN-224 and PCN-PPy variants based MOFtors.


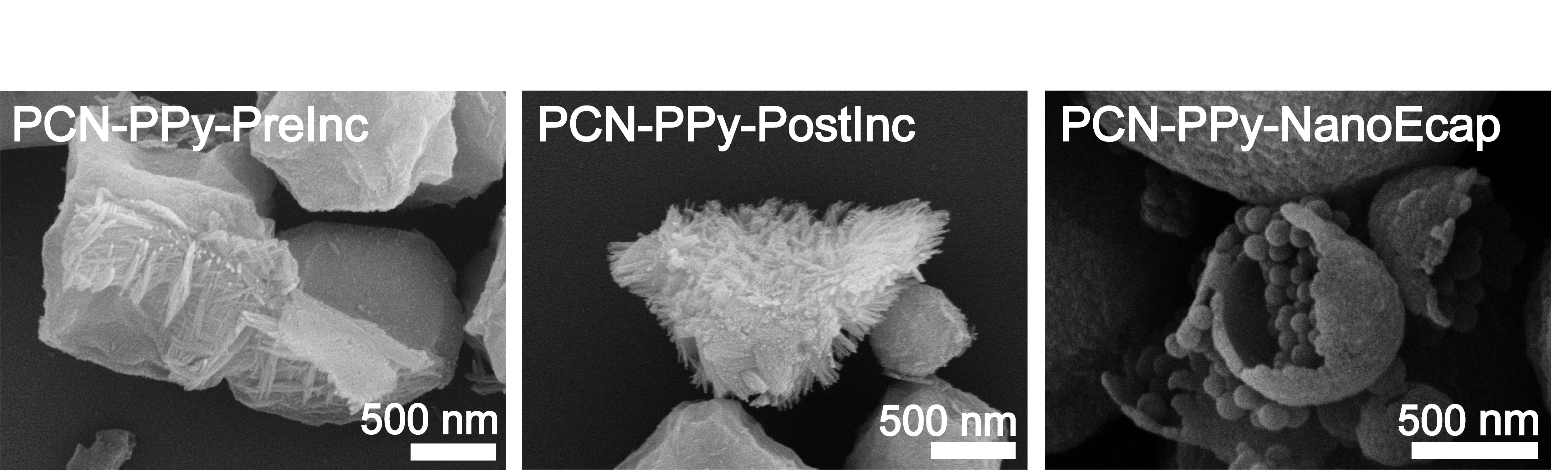


**Figure S2.** The cross-sectional scans of PCN-PPy variants based MOFtors.

**
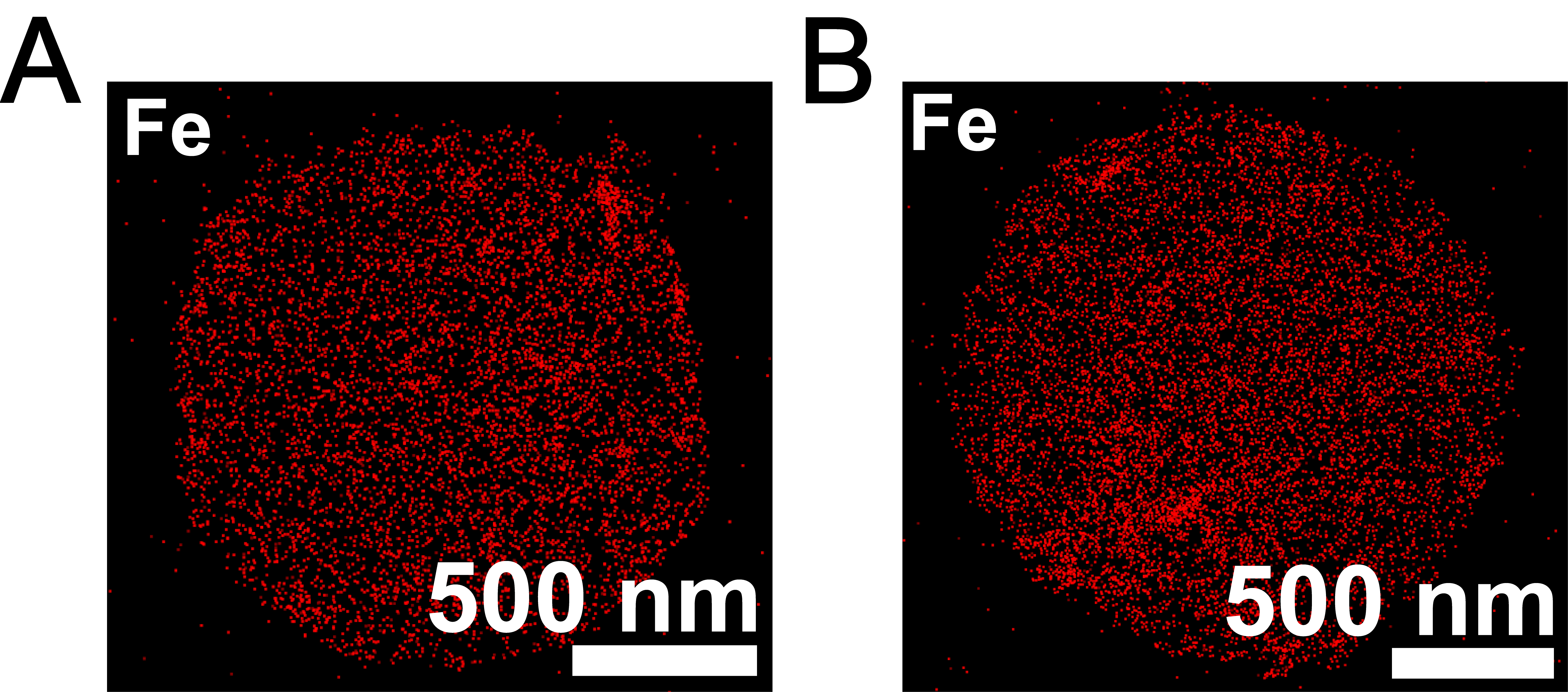
**

**Figure S3.** The Fe element distribution mapping images of (A) PCN-PPy-PreInc and (B) PCN-PPy-PostInc based MOFtors.

**
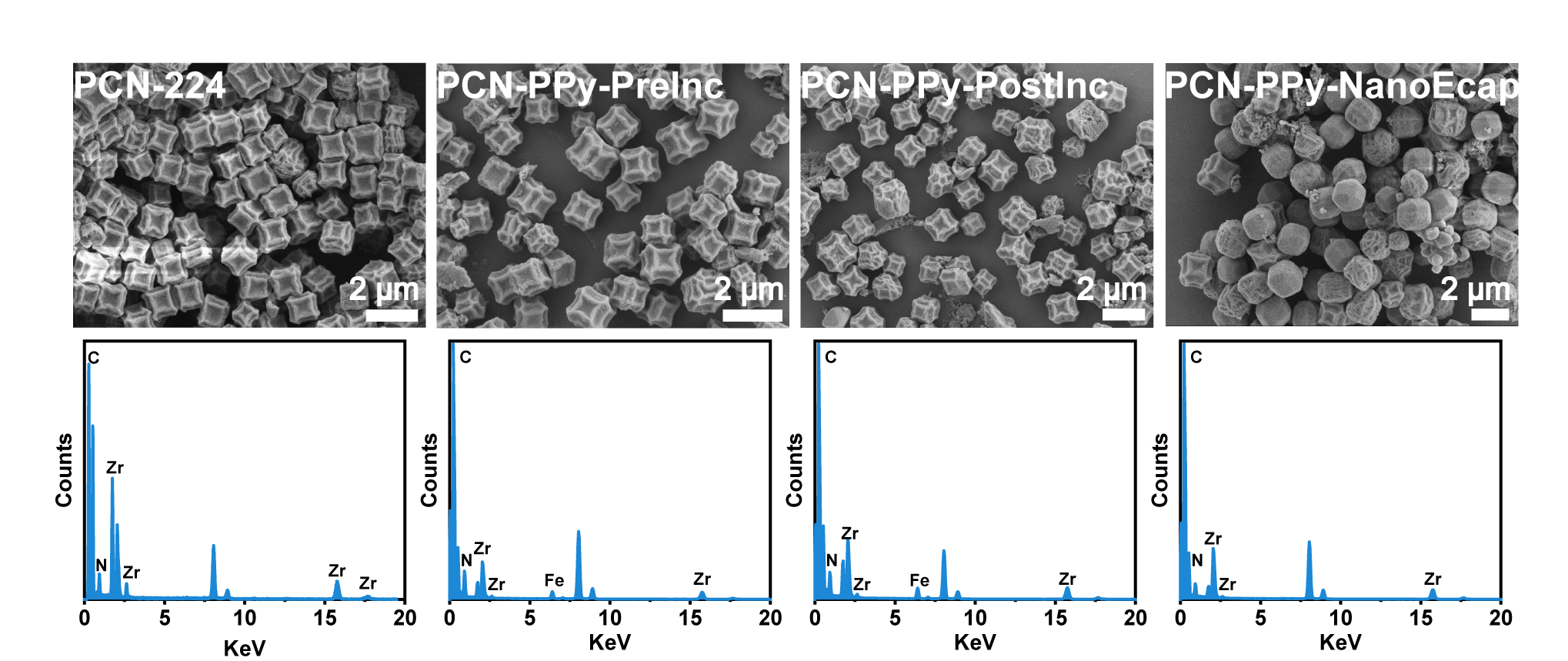
**

**Figure S4.** SEM images and the corresponding EDX spectra of the PCN-224 and PCN-PPy variants based MOFtors.

**
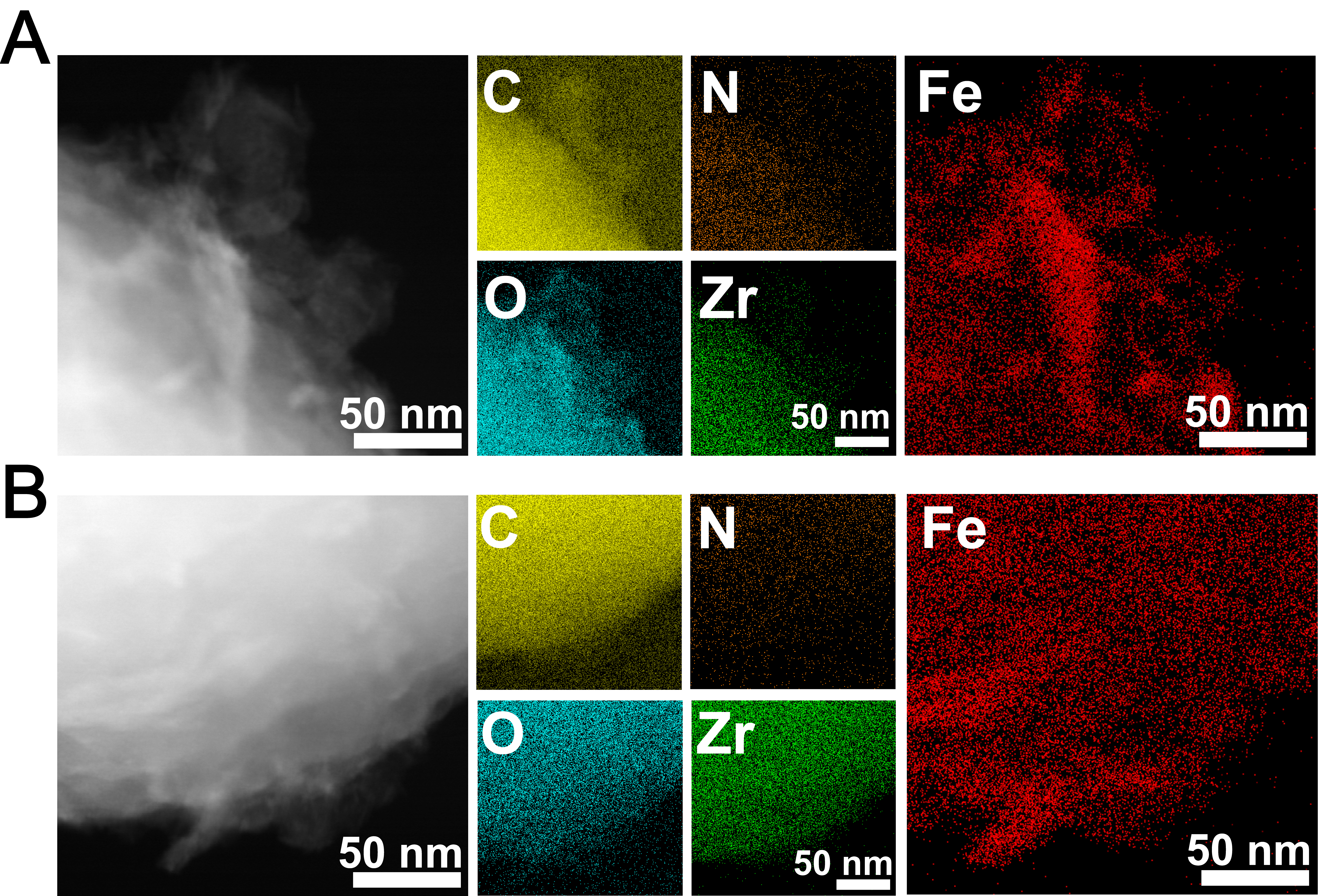
**

**Figure S5.** TEM and the corresponding elemental distribution mapping images of the PCN-224 and PCN-PPy variants based MOFtors near the surface. C atom (yellow), N atom (orange), O atom (cyan), and Zr atom (green).

**
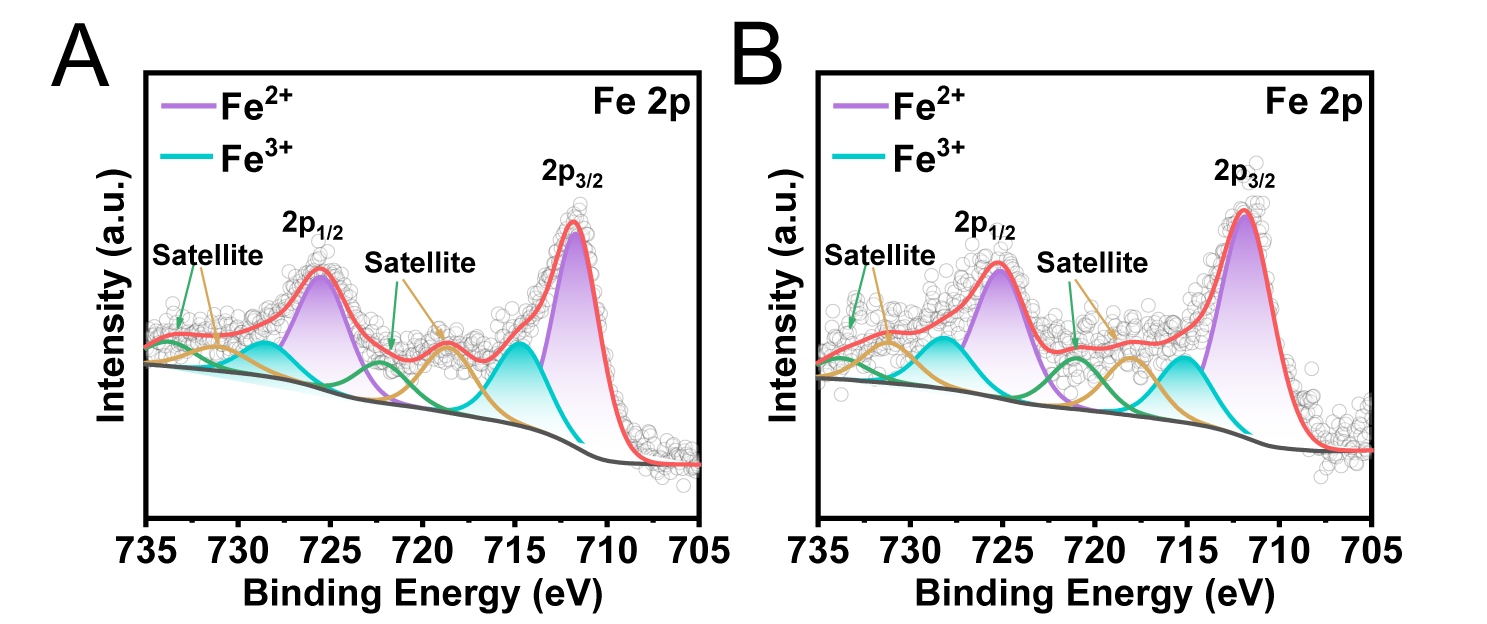
**

**Figure S6.** High-resolution XPS spectra of Fe 2p for (A) PCN-PPy-PreInc and (B) PCN-PPy-PostInc based MOFtors.


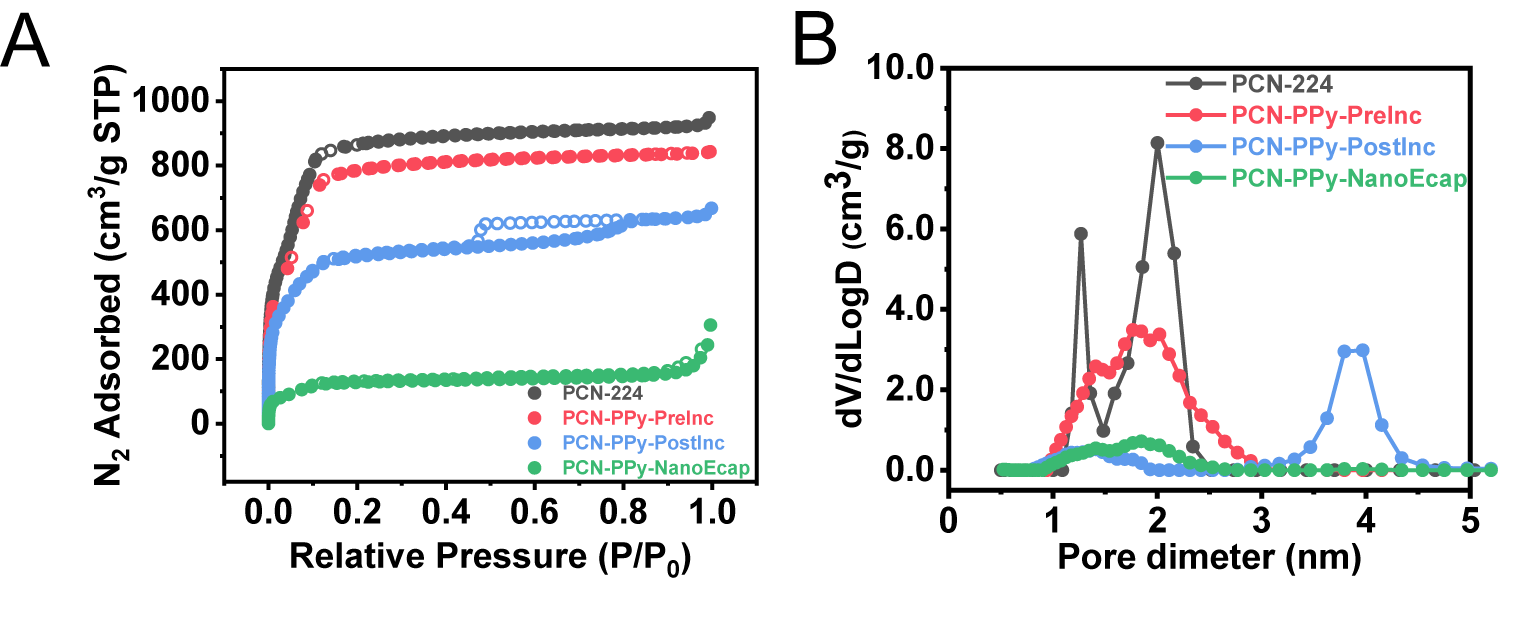


**Figure S7.** (A) N_2_ adsorption-desorption isotherms and (B) the corresponding pore size distribution of PCN-224 and PCN-PPy variants based MOFtors.

**Figure S8.** Velocities of PCN-PPy-PreInc based MOFtors under blue, UV, and NIR light irradiation of different power densities in a medium with different pH values.


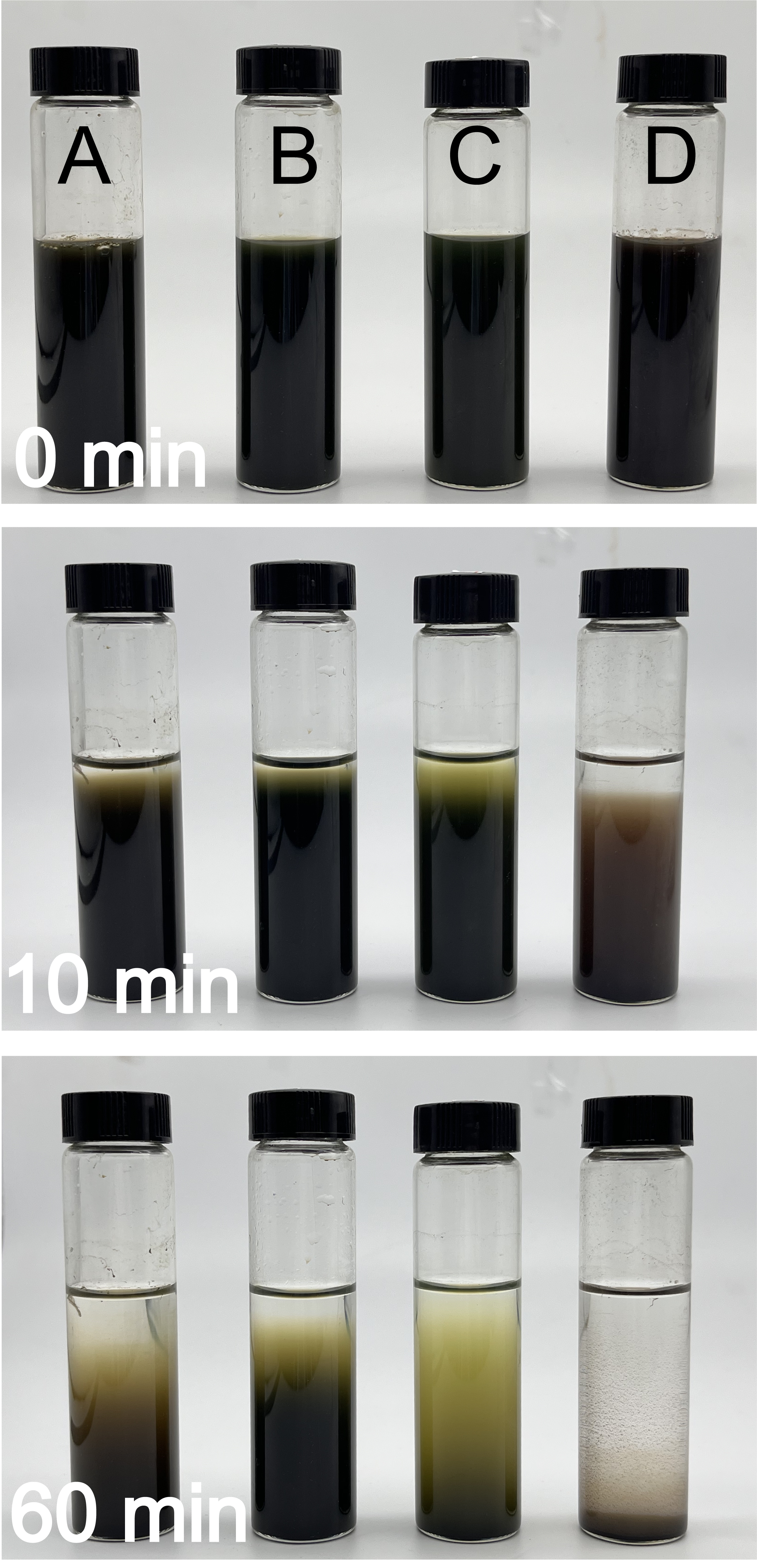


**Figure S9.** Time-lapse images of static settlement of A) PCN-224, B) PCN-PPy-PreInc, C) PCN-PPy-PostInc, D) PCN-PPy-NanoEcap based MOFtors.


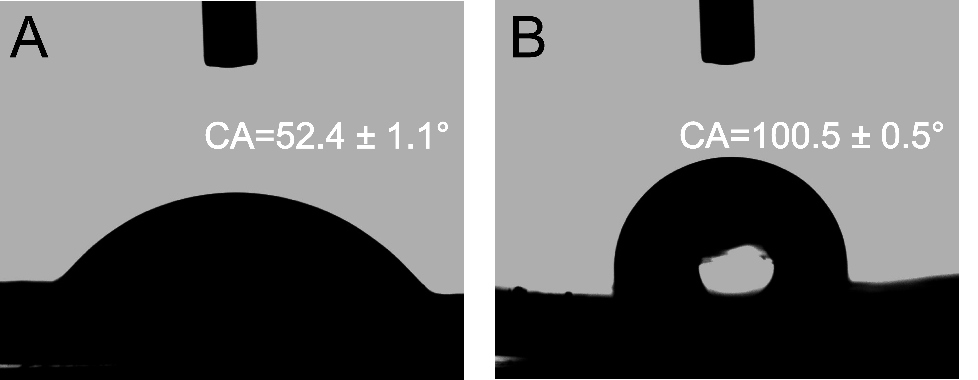


**Figure S10.**  Water contact angles of (A) PCN-224 and (B) PPy.

**
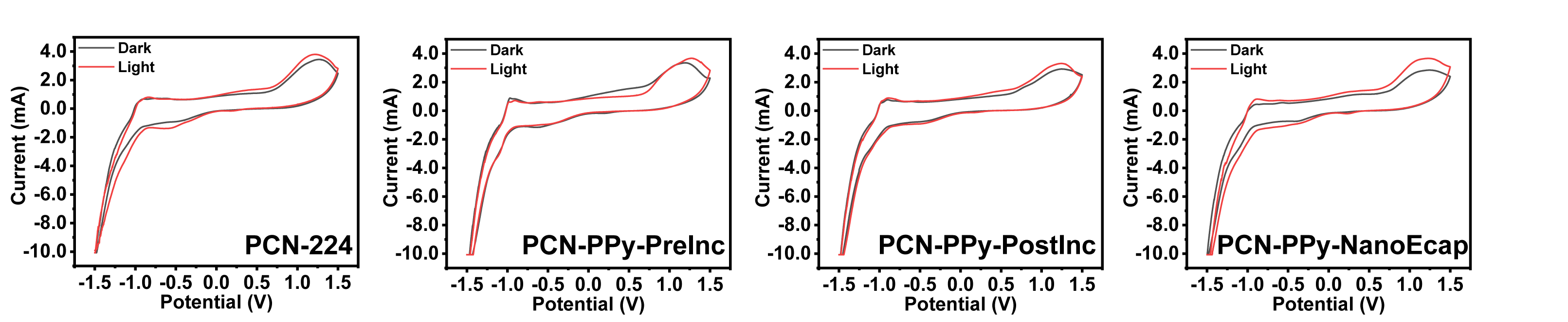
**

**Figure S11.** CV curves of PCN-224 and PCN-PPy variants based MOFtors in saturated 0.5 M Na_2_SO_4_ solution in the dark and under Xenon lamp irradiation.


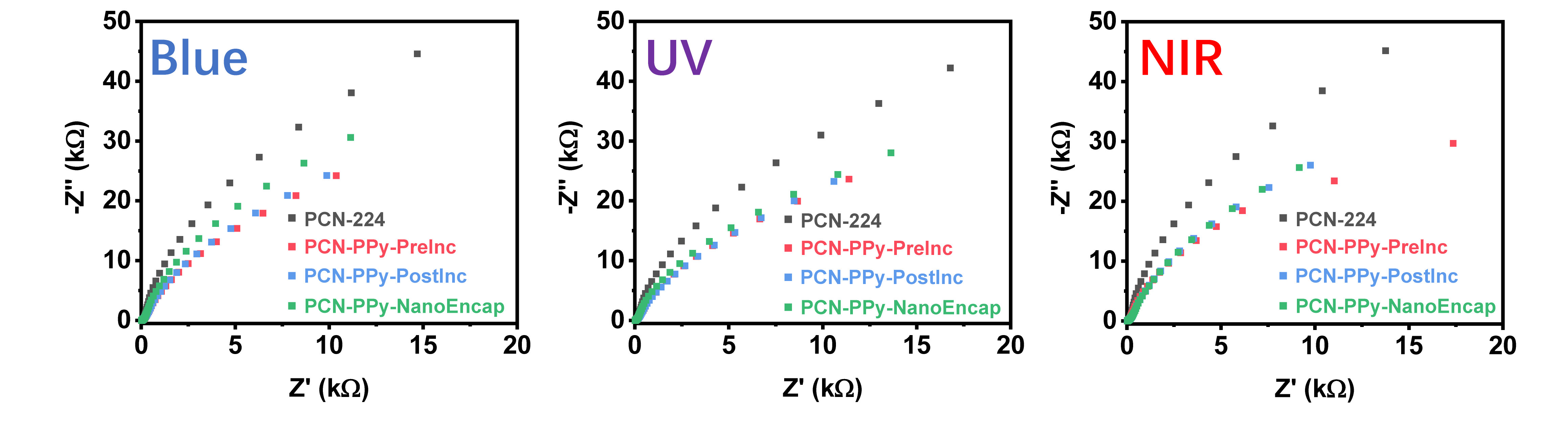


**Figure S12.** Electrochemical impedance spectra (EIS) of PCN-224 and PCN-PPy variants based MOFtors under irradiation with blue, UV, and NIR light.


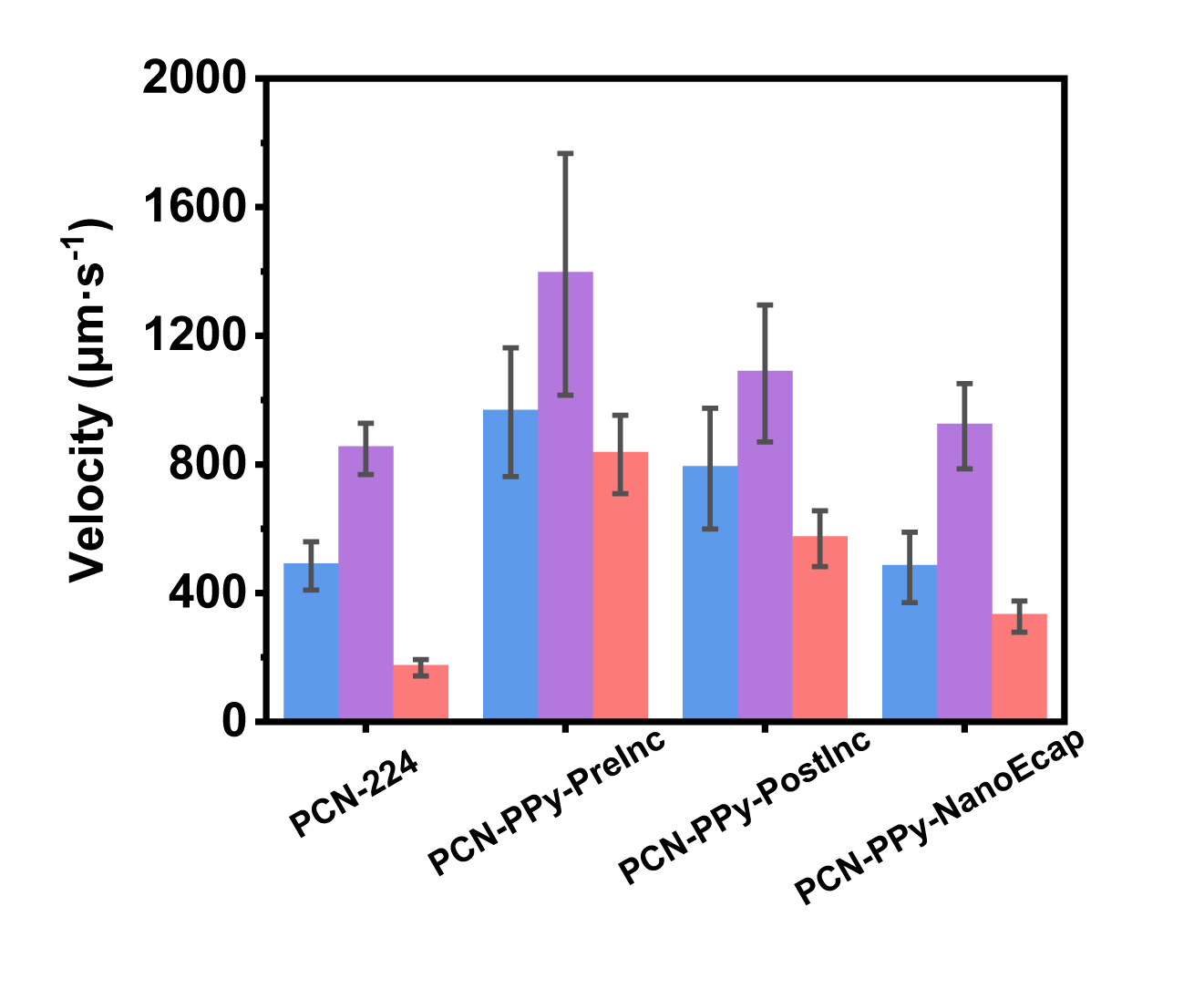


**Figure S13.** Velocities of PCN-224 and PCN-PPy variants based MOFtors under blue, UV, and NIR light irradiation of different power densities in 1M NaCl aqueous solution.


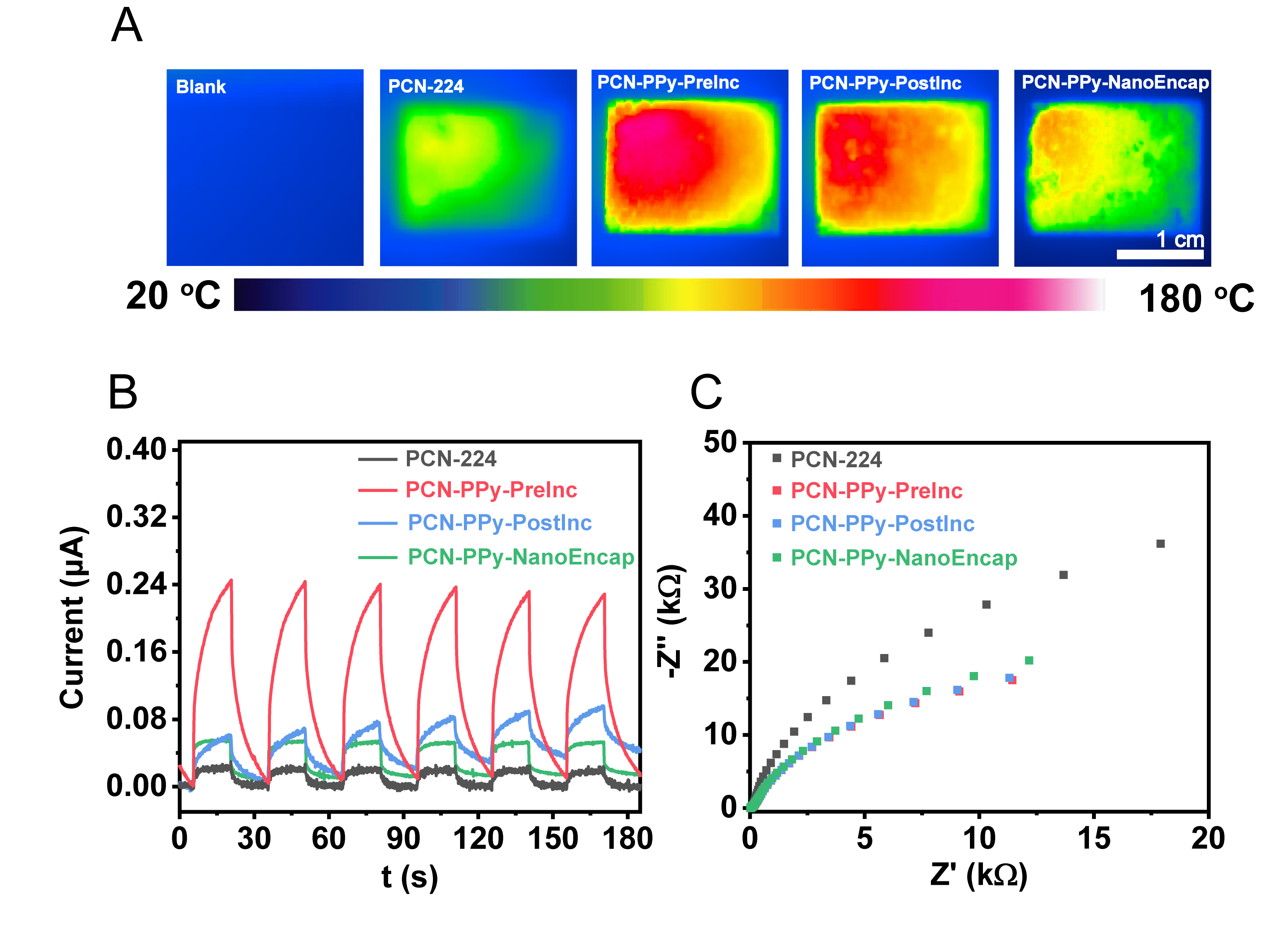


**Figure S14.** (A) Thermal imaging, (B) Transient photocurrent-time curves, (C) Electrochemical impedance spectra (EIS) of PCN-224 and PCN-PPy variants based MOFtors under Xenon lamp irradiation.


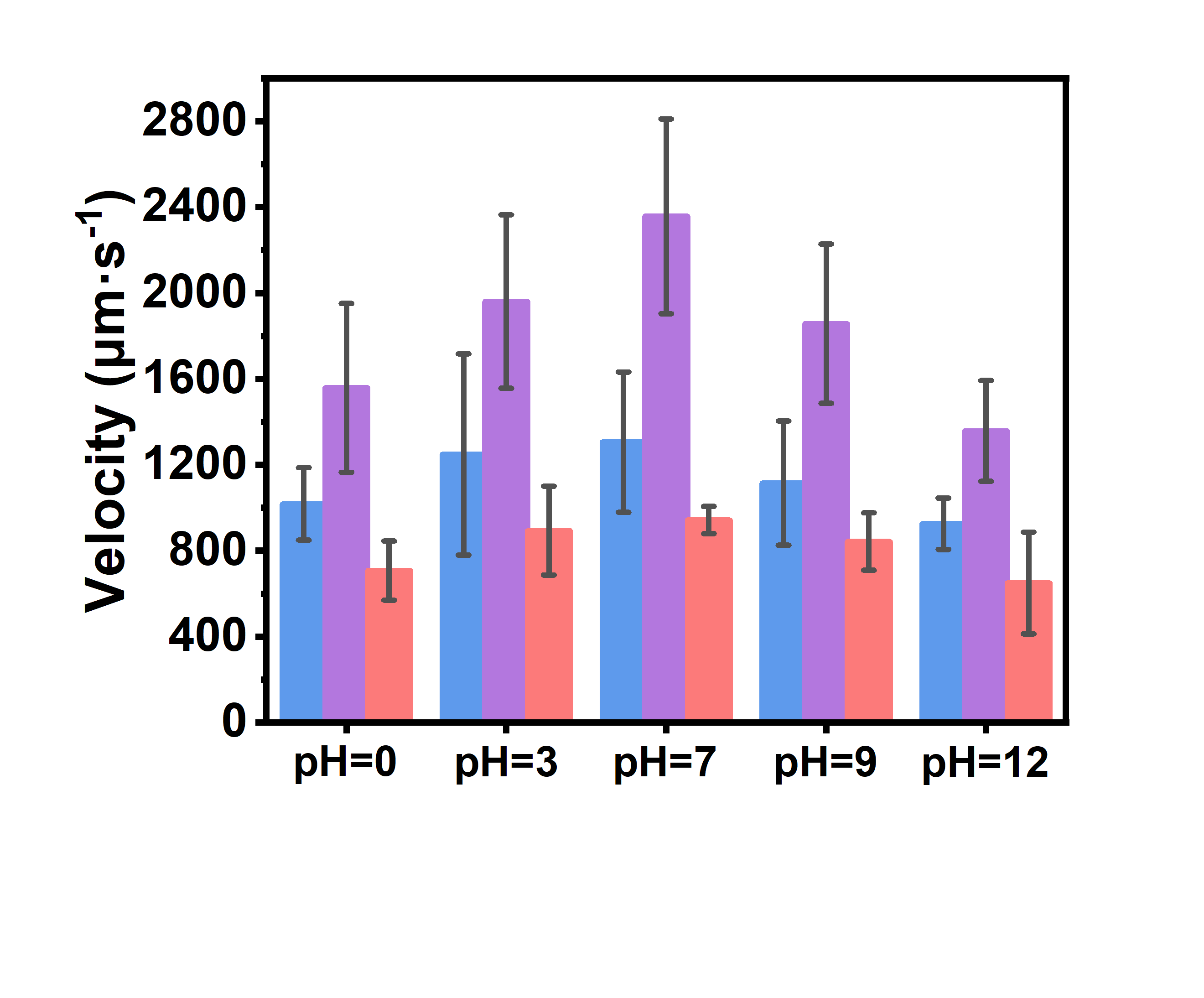


**Figure S15.** Velocities of PCN-224 and PCN-PPy variants based MOFtors under blue, UV, and NIR light irradiation of different power densities in solution with different pH values.


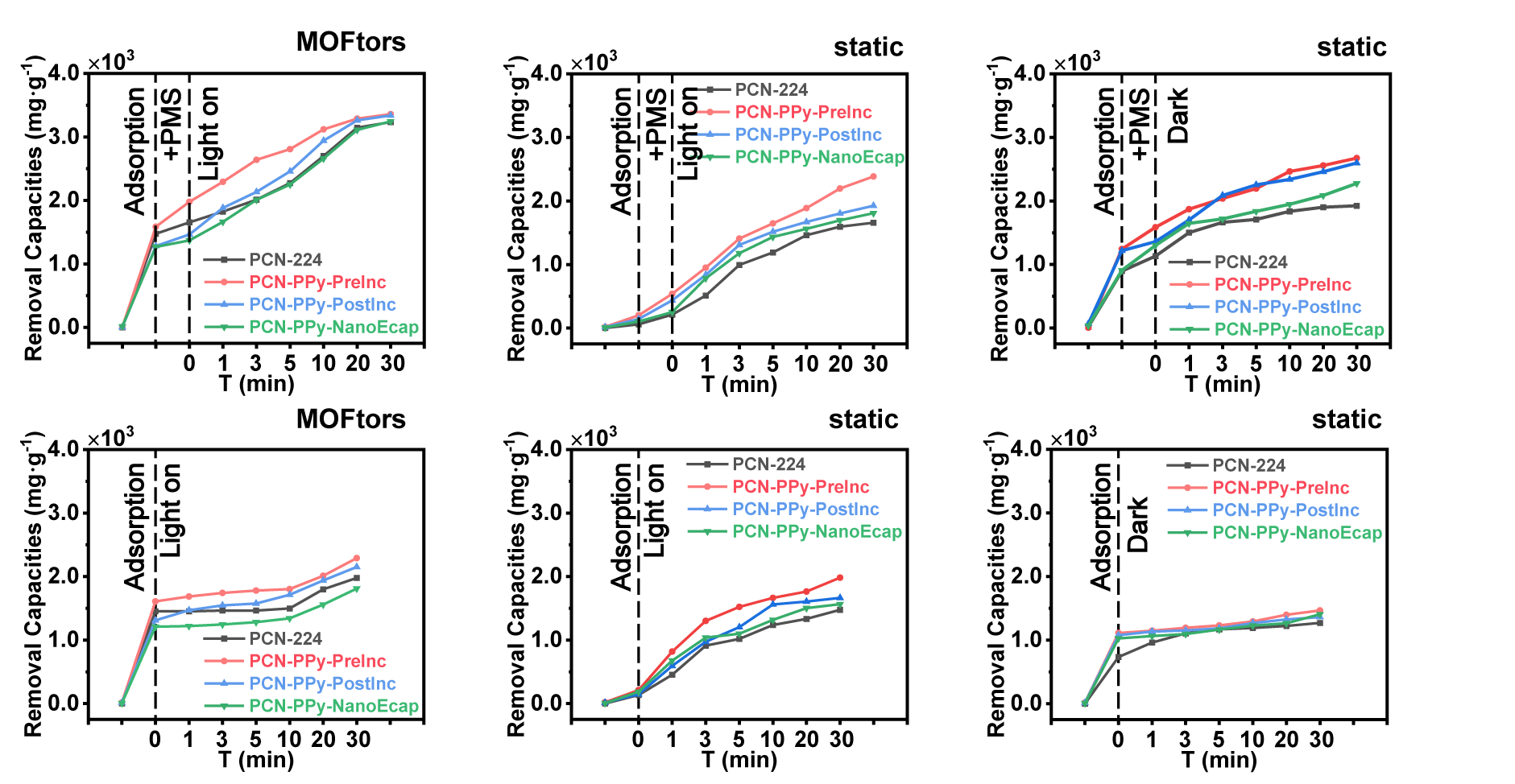


**Figure S16.** The line graphs of removal performance over time in relation to the removal of TCH under different conditions.


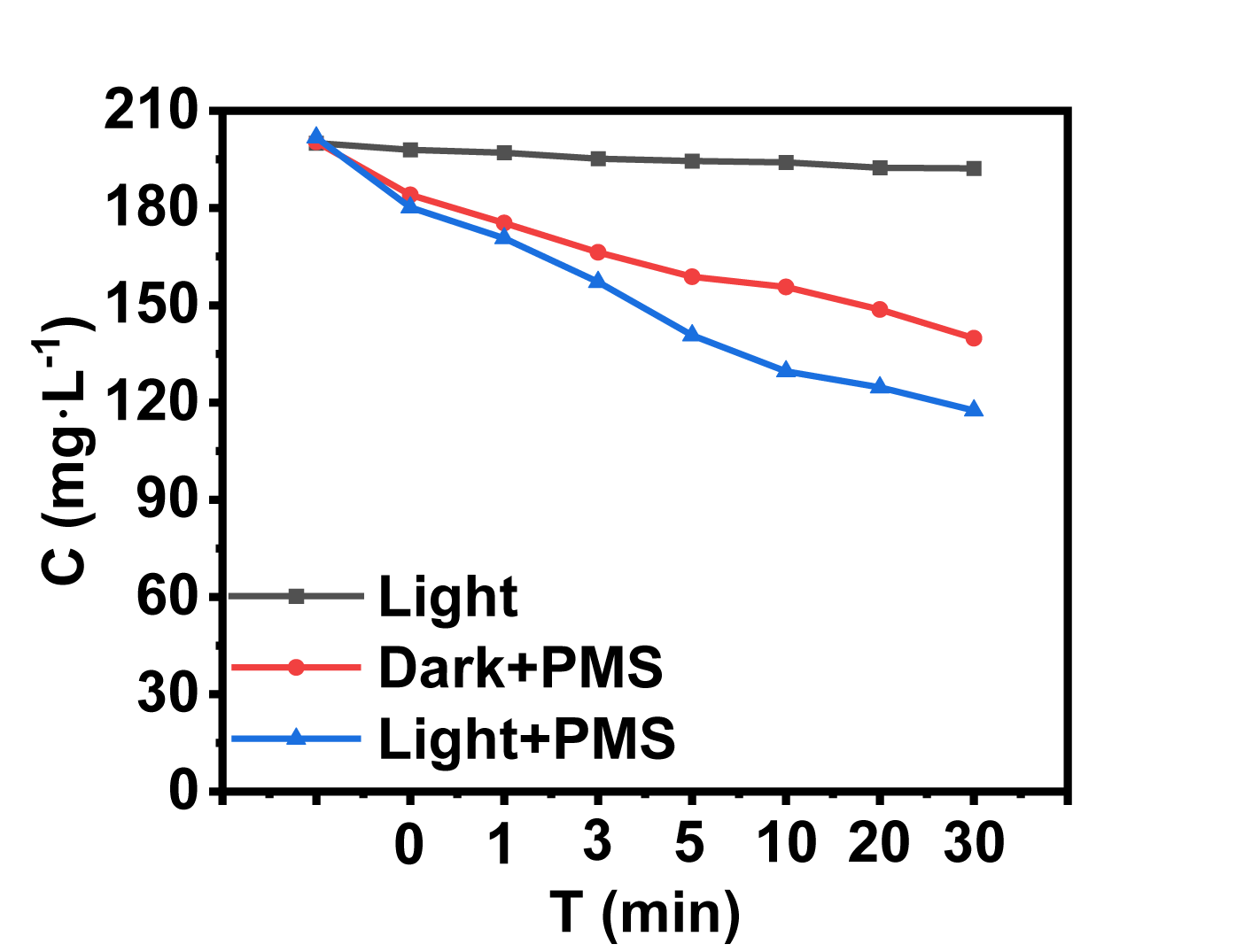


**Figure S17.** Timeline graphs of TCH removal under different conditions: solely Xe lamp, dark with PMS, and Xe lamp with PMS.

**
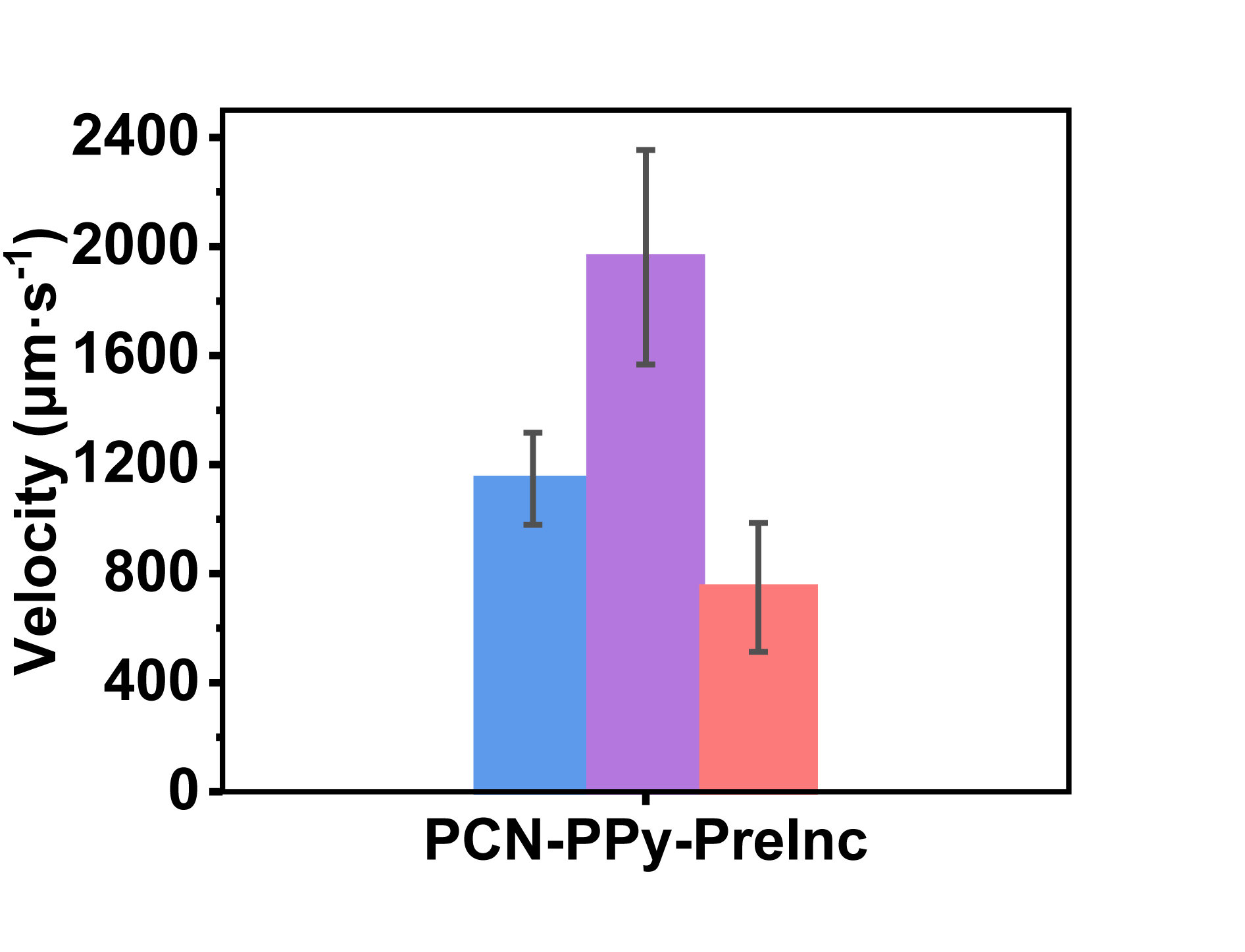
**

**Figure S18.** Velocities of PCN-PPy-PreInc based MOFtors under blue, UV, and NIR light irradiation of different power densities after 30min irradiation by Xenon lamp.

**
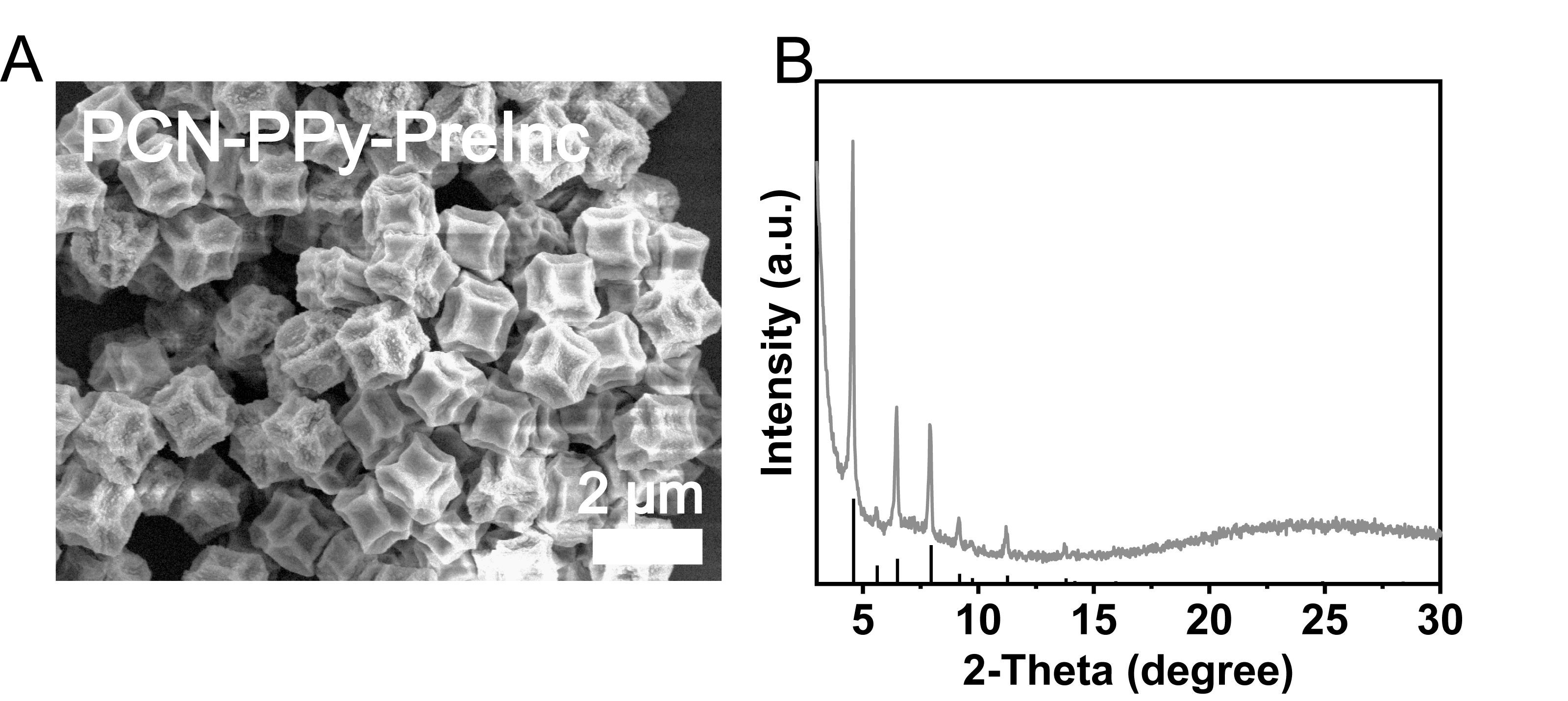
**

**Figure S19.** (A) SEM images and (B) XRD patterns of PCN-PPy-PreInc after five cycles.


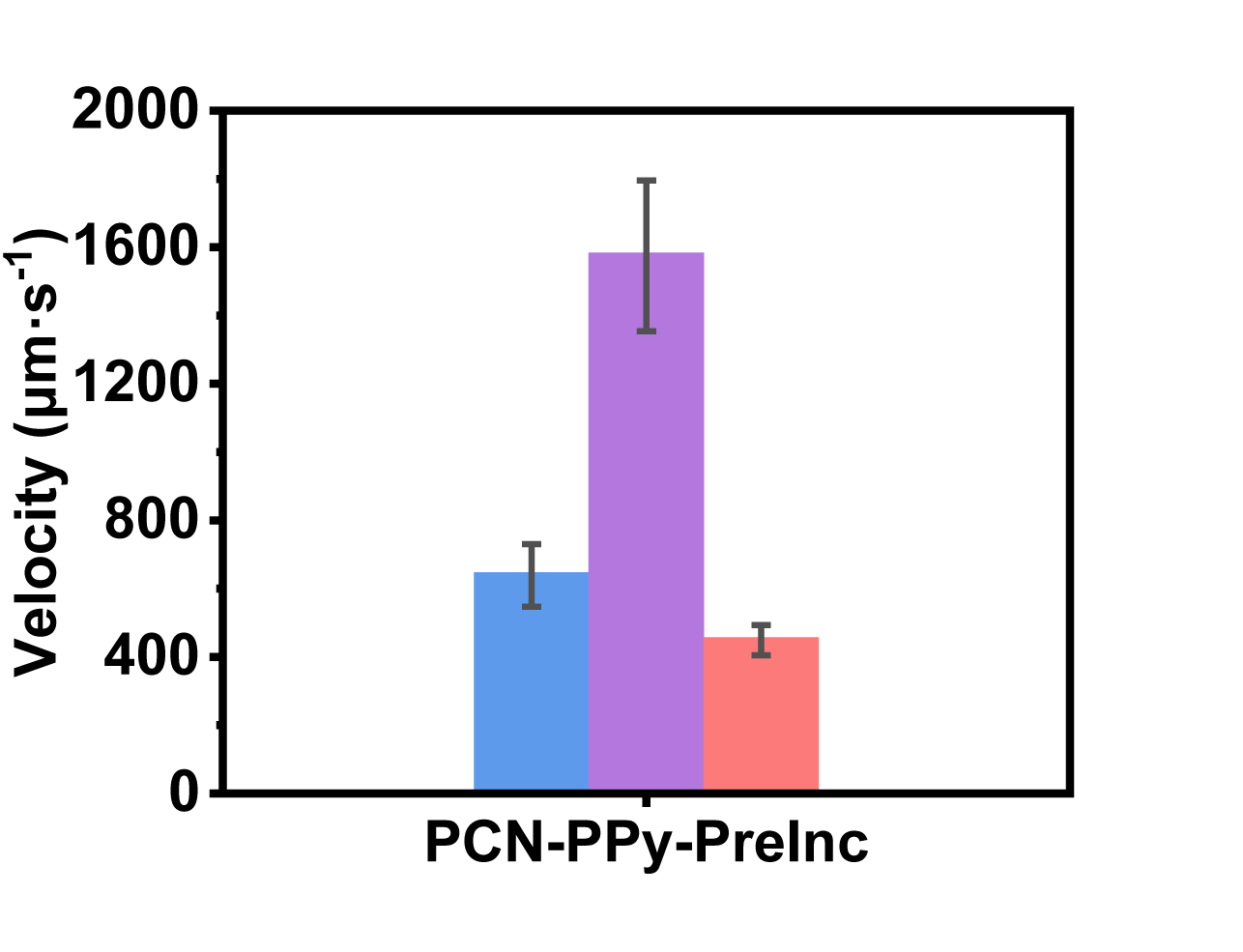


**Figure S20.** Velocities of PCN-PPy-PreInc based MOFtors under blue, UV, and NIR light irradiation of different power densities in a mixture of 1M NaCl and 1mM PMS.

**
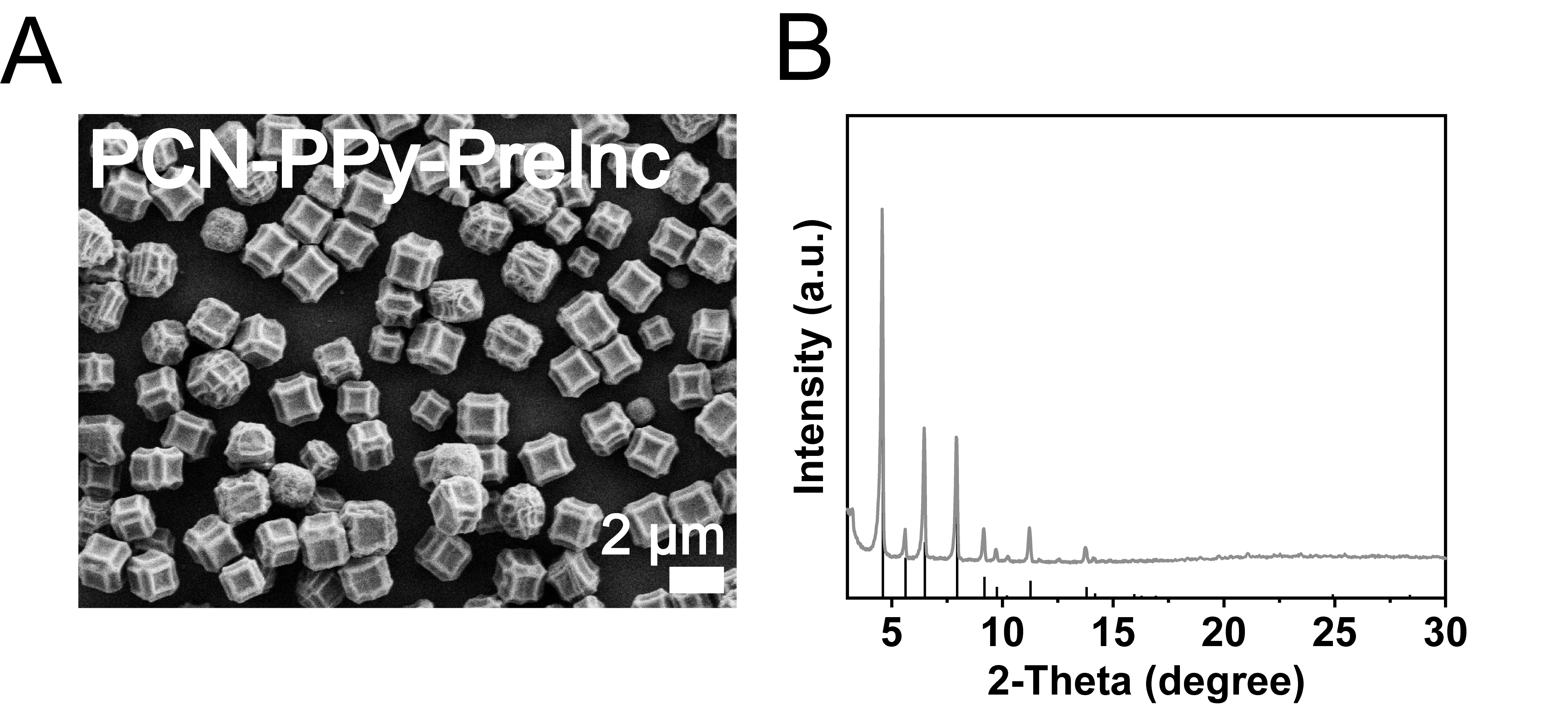
**

**Figure S21.** (A) SEM images and (B) XRD patterns of PCN-PPy-PreInc after five cycles.


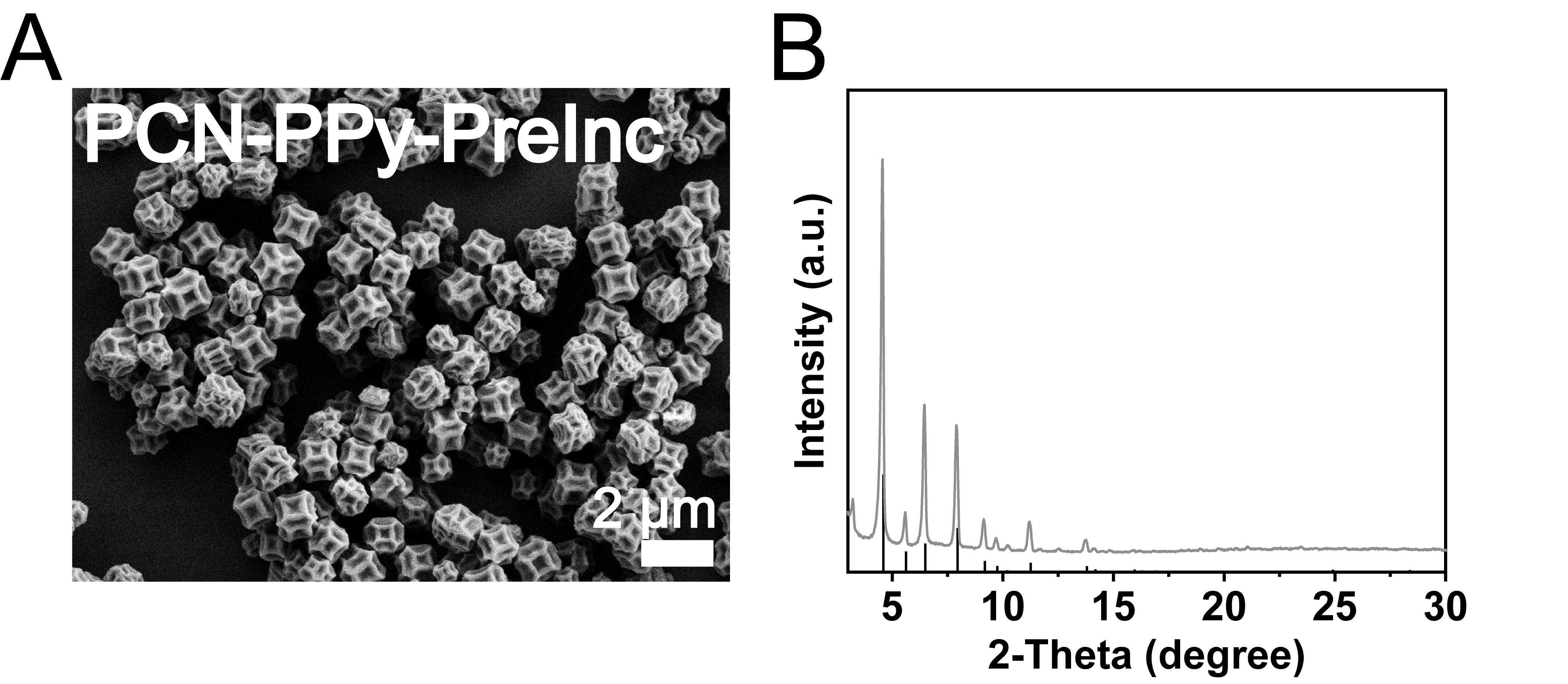


**Figure S22.** (A) SEM images and (B) XRD patterns of PCN-PPy-PreInc after being stored at room temperature for one month.


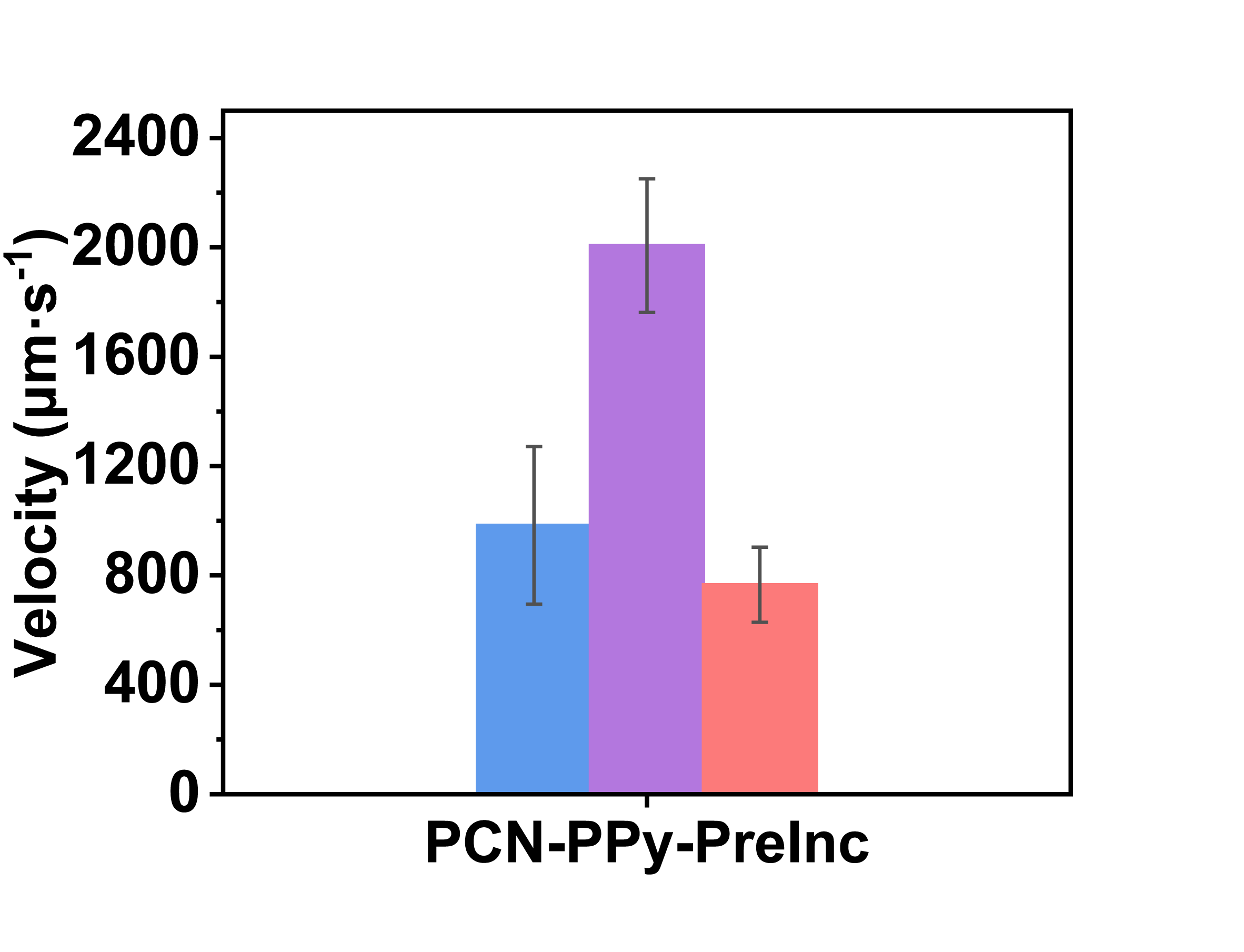


**Figure S23.** Velocities of PCN-PPy-PreInc based MOFtors under blue, UV, and NIR light irradiation of different power densities after being stored at room temperature for one month.

**
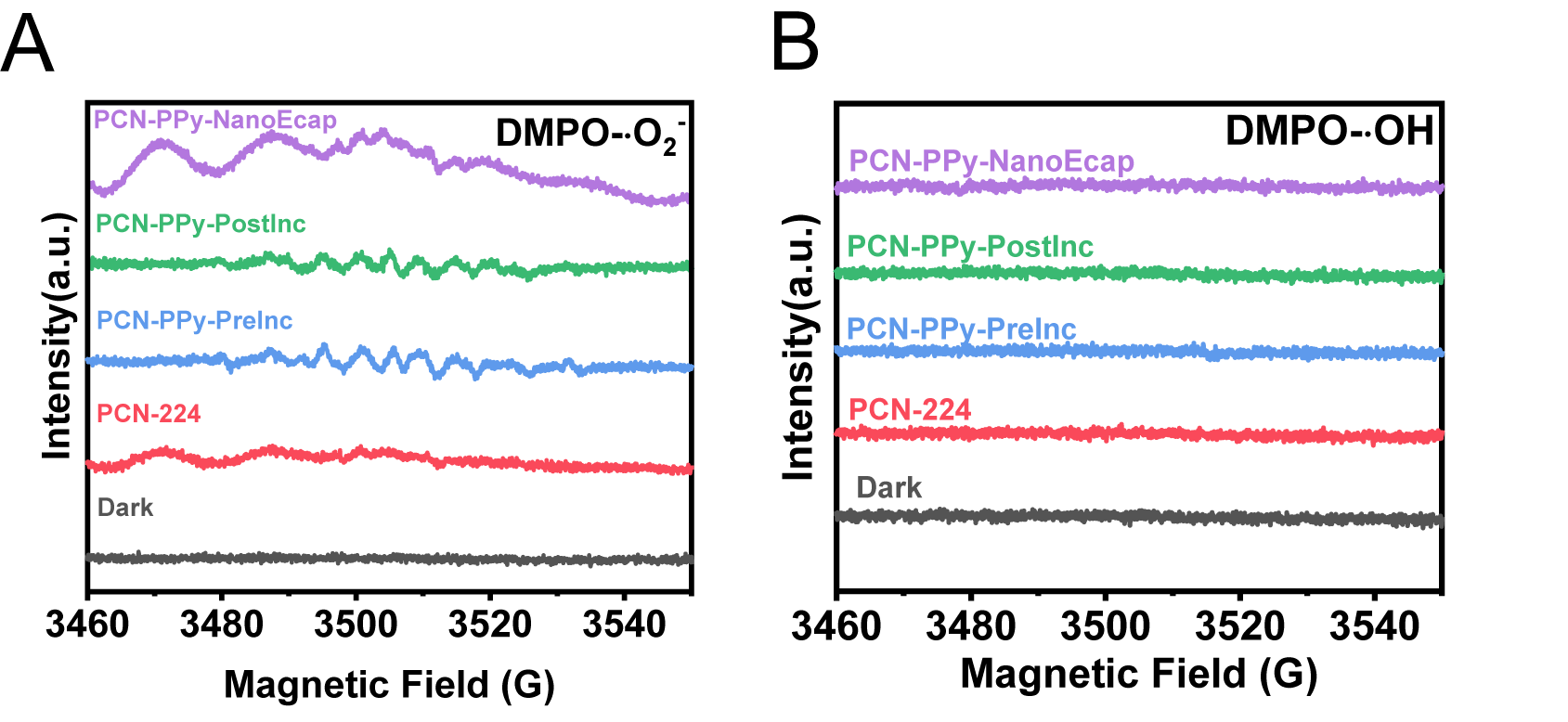
**

**Figure S24.** EPR Spectra of DMPO-·O_2_^-^ after adding PMS and DMPO-·OH without PMS under Xenon Lamp irradiation for PCN-224 and PCN-PPy variants.

**
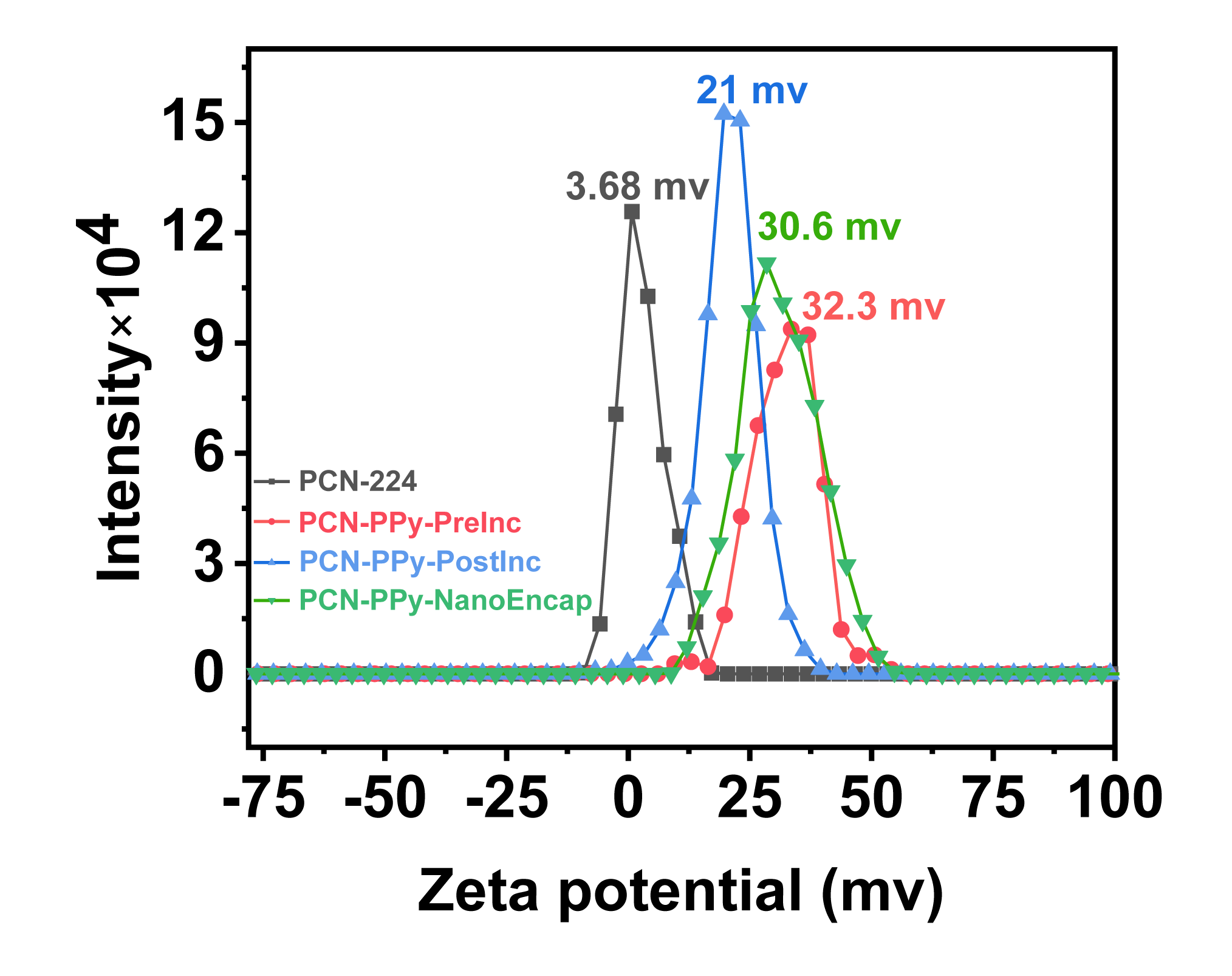
**

**Figure S25.** Zeta potential of PCN-224 and PCN-PPy variants based MOFtors.

**
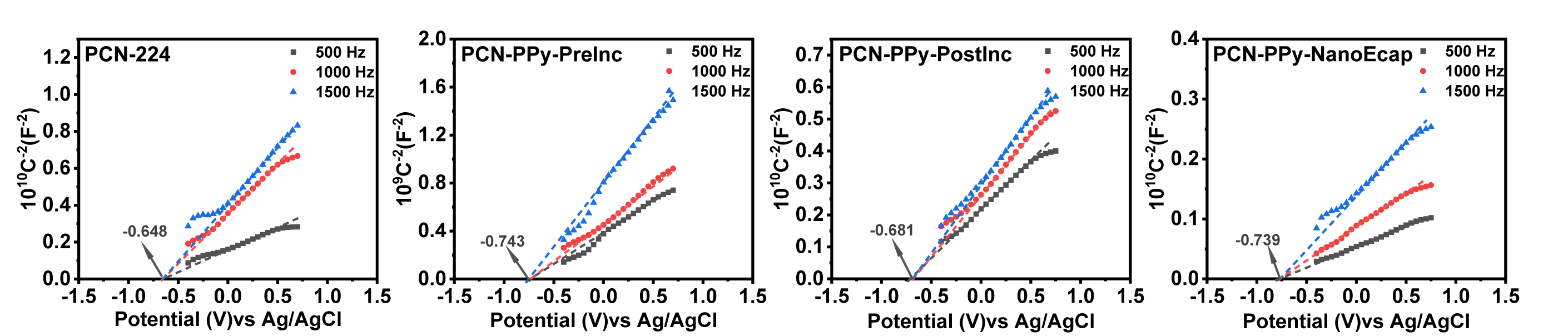
**

**Figure S26.** Motshottky curves of PCN-224 and PCN-PPy variants based MOFtors.


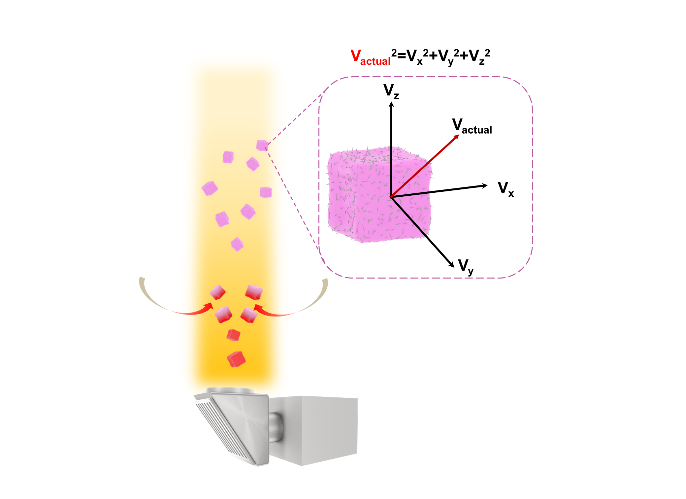


**Figure S27.** The motion speed calculation measurement of the MOFtors in the XY plane.

The average speed calculated in this work is the motion speed on the XY planes, i.e., V_xy_, due to the difficulty in the motion measurement in the Z direction **(Figure S27)**. In reality, the motion speed measurement of the micromotors should be in 3D space. Unfortunately, we could not perform it in our lab. For micron or submicron-sized motors, a conventional wide-field optical microscope is typically the primary instrument to visualize micro/nanomotors movement. However, the short depth-of-focus of the optical microscopy system, typically around 2-3 μm, creates a trade-off between the magnification and the depth-of-focus. It can create a challenge when attempting to track the motion of MNMs with vertical movement, as they may quickly move out of the ideal focus plane, resulting in a defocused image. Thus, we test the motion speed of micromotors on a plane (XY plane), i.e., V_xy_, while the actual speed value of 3D motion should be larger according to the mathematic relationship.

## Supplementary Tables

**Table S1.** Summary of the relative content of individual elements in PCN-224 and PCN-PPy variants.

| **Sample**  **Element (%)** | **PCN-224** | **PCN-PPy-PreInc** | **PCN-PPy-PostInc** | **PCN-PPy-NanoEcap** |
| --- | --- | --- | --- | --- |
| **C** | 67.79 | 65.25 | 67.75 | 67.23 |
| **N** | 4.83 | 5.71 | 4.94 | 6.29 |
| **O** | 23.98 | 24.35 | 23.42 | 23.51 |
| **Zr** | 3.4 | 3.56 | 2.99 | 2.97 |
| **Fe** | - | 1.13 | 0.90 | - |

**Table S2.** Summary of the relative content of three nitrogen signals in the N 1s spectra of PCN-224 and PCN-PPy variants.

| **Sample**  **Area (%)** | **PCN-224** | **PCN-PPy-PreInc** | **PCN-PPy-PostInc** | **PCN-PPy-NanoEcap** |
| --- | --- | --- | --- | --- |
| **-N+H-** | 11.2 | 12.2 | 12.8 | 13.0 |
| **C-N** | 56.1 | 60.9 | 61.4 | 62.2 |
| **C=N** | 32.7 | 26.9 | 25.8 | 24.8 |

**Table S3.** Temperatures (T, °C) of blank, PCN-224, and PCN-PPy variants under blue, UV, NIR light, and Xenon lamp irradiation for 1 min.

| **Sample**  **Light** | **Control** | **PCN-224** | **PCN-PPy-PreInc** | **PCN-PPy-PostInc** | **PCN-PPy-NanoEcap** |
| --- | --- | --- | --- | --- | --- |
| **Blue** | 32.6 | 83.3 | 101.6 | 91.1 | 88.1 |
| **UV** | 44.6 | 146.6 | 171.5 | 155.6 | 155.1 |
| **NIR** | 31.5 | 47.9 | 55.5 | 49.8 | 68.1 |
| **Xe Lamp** | 33.2 | 101.5 | 150.4 | 143.8 | 121.7 |

**Table S4.** Comparison of properties of different adsorbents, catalysts, adsorbent catalysts and micromotors, and PMS activation and removal of TCH.

|  | **Sample** | **Dosage**  **(g/L)** | **TCH**  **(mg/L)** | **PMS**  **(mM)** | **Removal rate**  **(%)** | **Irradiation time**  **(min)** | **Removal Capacities**  **(mg/g)** | **Ref** |
| --- | --- | --- | --- | --- | --- | --- | --- | --- |
| **Adsorption** | **ZIF-8P3(4)** | 0.10 | 100 | - | 97.68 | 60 | 976.80 | ^[2]^ |
|  | **Co-MIL-300** | 0.60 | 280 | - | 57.3 | 25 | 267.30 | ^[3]^ |
| **Catalytic** | **HCNFs** | 0.20 | 50 | 0.80 | 87.50 | 120 | 218.75 | ^[4]^ |
|  | **C-Co-TN** | 0.10 | 20 | 0.75 | 97.40 | 80 | 194.80 | ^[5]^ |
|  | **Co@NCNTs-600** | 0.12 | 20 | 2.00 | 93.10 | 20 | 155.17 | ^[6]^ |
|  | **55FeCoO_x_/CN-Vn** | 0.04 | 25 | 1.00 | 91.50 | 12 | 571.88 | ^[7]^ |
|  | **Bi/BiVO_4_-CdS** | 0.40 | 20 | - | 85.50 | 30 | 42.75 | ^[8]^ |
|  | **BiOBr** | 0.20 | 20 | - | 96.50 | 60 | 96.50 | ^[9]^ |
|  | **e-BN/e-CN** | 1.00 | 40 | - | 91.00 | 60 | 36.40 | ^[10]^ |
|  | **NiCo_2_O_4_/g-C_3_N_4_-N_vac_** | 0.10 | 25 | 1.00 | 97.40 | 30 | 243.50 | ^[11]^ |
|  | **MnCo_2_O_4_/g-C_3_N_4_** | 0.04 | 20 | 1.00 | 96.10 | 12 | 480.50 | ^[12]^ |
| **Adsorption+ Catalytic**  **+ Catalytic** | **YCDs@UiO-66** | 0.30 | 20 | - | 92.60 | 60 | 61.73 | ^[13]^ |
|  | **ZnIn_2_S_4_@PCN-224** | 0.20 | 20 | - | 99.90 | 60 | 99.90 | ^[14]^ |
|  | **2Cl-MIL-53** | 0.10 | 25 | - | 82.30 | 16 | 205.75 | ^[15]^ |
|  | **UML-2** | 1.00 | 20 | - | 91.80 | 60 | 18.36 | ^[16]^ |
|  | **Bi_2_WO_6_/Bi-MOF** | 0.10 | 10 | - | 60.00 | 30 | 60.00 | ^[17]^ |
|  | **15NiCo-DU50** | 0.30 | 20 | 0.40 | 98.50 | 8 | 65.67 | ^[18]^ |
|  | **Co-ZIF-(Fe)0.5** | 0.20 | 30 | 0.67 | 96.71 | 30 | 145.07 | ^[19]^ |
|  | **Zn/Co-MOF@rGO-600** | 0.05 | 44.5 | 0.50 | 91.66 | 60 | 815.77 | ^[20]^ |
| **Micromotors** | **Pt-ZIS micromotors** | 0.27 | 30 | - | 70.00 | 180 | 78.65 | ^[21]^ |
|  | **FMO micromotors** | 0.3 | 50 | - | 90.00 | 180 | 150.00 | ^[22]^ |
|  | **CuS@Fe_3_O_4_/Pt micromotors** | 0.8 | 40 | - | 78.00 | 30 | 39.00 | ^[23]^ |
|  | **PASP/Fe_2_O_3_-MnO_2_ micromotors** | 2 | 30 | - | 90.00 | 50 | 13.50 | ^[24]^ |
|  | **PCN-PPy-PreInc** | 0.05 | 200 | 0.10 | 83.70 | 30 | 3347.30 | **This work** |

**Table S5.** The proposed intermediates of TCH degradation in the PCN-PPy-PreInc based MOFtors /PMS/Light system.

| **Compound** | **Molecular formula** | **m/z** | **Possible structure** |
| --- | --- | --- | --- |
| **TCH** | C_22_H_24_O_8_N_2_ | 445 | 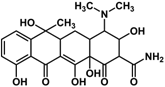 |
| **P1** | C_22_H_24_O_9_N_2_ | 461 | 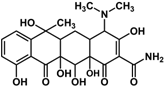 |
| **P2** | C_20_H_22_O_10_N_2_ | 451 | 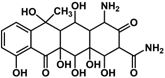 |
| **P3** | C_21_H_20_O_7_N_2_ | 413 | 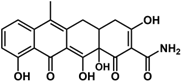 |
| **P4** | C_20_H_19_O_8_ | 386 | 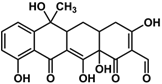 |
| **P5** | C_20_H_17_O_13_N | 480 | 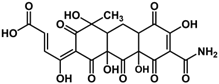 |
| **P6** | C_18_H_17_O_3_N | 296 | 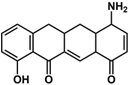 |
| **P7** | C_13_H_18_O | 191 | 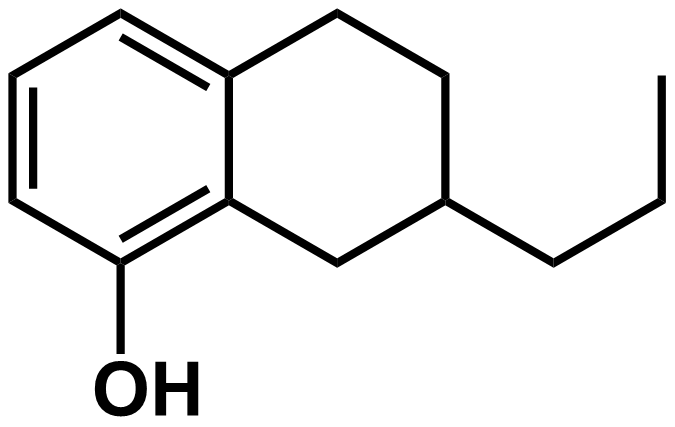 |
| **P8** | C_14_H_18_O_4_ | 250 | 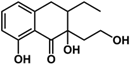 |
| **P9** | C_16_H_17_O_5_ | 290 | 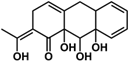 |
| **P10** | C_11_H_19_ON | 182 | 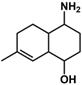 |
| **P11** | C_7_H_6_O_2_ | 123 | 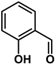 |
| **P12** | C_10_H_10_O_2_ | 162 | 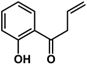 |
| **P13** | C_12_H_21_O_3_ | 213 | 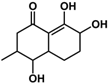 |

## References

[1] D. Feng, W.-C. Chung, Z. Wei, Z.-Y. Gu, H.-L. Jiang, Y.-P. Chen, D. J. Darensbourg, H.-C. Zhou *J. Am. Chem. Soc.* **2013,** *135,* 17105-17110.

[2] Z. Zhang, Y. Chen, C. Hu, C. Zuo, P. Wang, W. Chen, T. Ao *Environ. Res.* **2021,** *198,* 111254.

[3] L. Nie, Y. Yang, C. Fang, H. Chen, S. Xin *Appl. Surf. Sci.* **2023,** *640,* 158390.

[4] C. Wang, J. Kim, M. Kim, H. Lim, M. Zhang, J. You, J.-H. Yun, Y. Bando, J. Li, Y. Yamauchi *J. Mater. Chem. A* **2019,** *7,* 13743-13750.

[5] Q. Li, J. Liu, Z. Ren, Z. Wang, F. Mao, H. Wu, R. Zhou, Y. Bu *Chem. Eng. J.* **2022,** *429,* 132269.

[6] L. Hu, Y. Zhang, X. Liu, H. Zhu, J. Wu, Y. Wang, Y. Long, G. Fan *Chem. Eng. J.* **2022,** *450,* 138219.

[7] J. Jiang, X. Wang, C. Yue, T. Li, M. Li, C. Li, S. Dong *Chem. Eng. J.* **2022,** *427,* 131702.

[8] Y. Xue, Z. Chen, Z. Wu, F. Tian, B. Yu *Sep. Purif. Technol.* **2021,** *275,* 119152.

[9] G. Chen, N. Hing Wong, J. Sunarso, Y. Wang, X. Huang, X. Xiong, F. Wang *Appl. Surf. Sci.* **2021,** *569,* 151011.

[10] L. Acharya, S. P. Pattnaik, A. Behera, R. Acharya, K. Parida *Inorg. Chem.* **2021,** *60,* 5021-5033.

[11] J. Jiang, X. Wang, C. Yue, S. Liu, Y. Lin, T. Xie, S. Dong *J. Hazard. Mater.* **2021,** *414,* 125528.

[12] X. Wang, J. Jiang, Y. Ma, Y. Song, T. Li, S. Dong *J. Colloid Interface Sci.* **2021,** *600,* 449-462.

[13] X. Wang, F. Yan, Y. Chen, X. Bai, Y. Fu *Chemosphere* **2023,** *343,* 140250.

[14] P. Jin, L. Wang, X. Ma, R. Lian, J. Huang, H. She, M. Zhang, Q. Wang *Appl. Catal. B* **2021,** *284,* 119762.

[15] X. Wang, Y. Ma, J. Jiang, M. Li, T. Li, C. Li, S. Dong *J. Hazard. Mater.* **2022,** *434,* 128864.

[16] S. P. Tripathy, S. Subudhi, A. Ray, P. Behera, J. Panda, S. Dash, K. Parida *J. Colloid Interface Sci.* **2023,** *629,* 705-718.

[17] Y. Yang, M. Xu, L. Ai, N. Guo, C. Leng, C. Tan, M. Lu, L. Wang, L. Huang, D. Jia *J. Environ. Chem. Eng.* **2023,** *11,* 109873.

[18] Y. Wang, C. Liu, C. Wang, Q. Hu, L. Ding *Chemosphere* **2022,** *299,* 134322.

[19] Q. Hu, J. Cao, Z. Yang, W. Xiong, Z. Xu, P. Song, M. Jia, Y. Zhang, H. Peng, A. Wu *Sep. Purif. Technol.* **2021,** *259,* 118059.

[20] M. Qi, P. Lin, Q. Shi, H. Bai, H. Zhang, W. Zhu *Process Saf. Environ. Protect.* **2023,** *171,* 847-858.

[21] M. Yuan, M. Gong, H. Huang, Y. Zhao, Y. Ying, S. Wang *Inorg. Chem. Front.* **2022,** *9,* 5725-5734.

[22] H. Ye, S. Wang, Y. Wang, P. Guo, L. Wang, C. Zhao, S. Chen, Y. Chen, H. Sun, S. Wang, X. Ma *Appl. Catal. B* **2022,** *314,* 121484.

[23] E. Ma, K. Wang, Z. Hu, H. Wang *J. Colloid Interface Sci.* **2021,** *603,* 685-694.

[24] X. Ding, Y. Liu, X. Chen, W. Liu, J. Li *Chem.-Asian J.* **2021,** *16,* 1930-1936.

**Supporting Videos:**

Video S1: Microscopic motion behavior of PCN-224 with different concentrations under different light with different power densities.

Video S2: Microscopic motion behavior of PCN-PPy-PreInc with different concentrations under different light with different power densities.

Video S3: Microscopic motion behavior of PCN-PPy-PostInc with different concentrations under different light with different power densities.

Video S4: Microscopic motion behavior of PCN-PPy-NanoEcap with different concentrations under different light with different power densities.

Video S5: Microscopic motion behavior of Polystyrene microspheres with a concentration of 2.0 mg·mL^−1^ under different light with different power densities.

Video S6: Microscopic motion behavior of PCN-224 and PCN-PPy variants in 1 M NaCl solution under different light with different power densities.

Video S7: Macroscopic motion behavior of PCN-224 and PCN-PPy variants under Xenon lamp with a power density of 400 mW cm^−2^ in a bottle with 30 mL water.

Video S8: Microscopic motion behavior of PCN-224 and PCN-PPy variants in 1 mM PMS solution under different light with different power densities.

Video S9: Microscopic motion behavior of PCN-PPy-PreInc after 30 min irradiation by Xenon lamp under different light with different power densities.

Video S10: Microscopic motion behavior of PCN-PPy-PreInc in a mixture of 1 M NaCl and 1 mM PMS under different light with different power densities.

Video S11: Microscopic motion behavior of PCN-PPy-PreInc after being stored at room temperature for one month under different light with different power densities.
